# Patent landscape analysis for materials based on fungal mycelium: a guidance report on how to interpret the current patent situation

**DOI:** 10.1186/s40694-024-00177-2

**Published:** 2024-08-10

**Authors:** Vera Meyer, Sabine Mengel

**Affiliations:** 1 Chair of Applied and Molecular Microbiology, Institute of Biotechnology, Technische Universität Berlin, Berlin, Germany; 2 Viering, Jentschura & Partner mbB, Grillparzerstr. 14, 81675 Munich, Germany

**Keywords:** Filamentous fungi, Fungal composite material, Biomaterial, Fungal leather, Textile, Packaging material

## Abstract

**Background:**

Recent advancements in the collaboration between two scientific disciplines—fungal biotechnology and materials sciences—underscore the potential of fungal mycelium as renewable resource for sustainable biomaterials that can be harnessed in different industries. As fungal mycelium can be biotechnologically obtained from different filamentous fungi and is as a material very versatile, respective research and commercial application should be thriving. However, some granted patents in the field of fungal mycelium-based materials have caused uncertainty in the community as to which subject matter is patent-protected and for how long the protection is expected to last.

**Results:**

This opinion paper therefore maps the patent landscape of fungal mycelium-based materials with a specific focus on technical applications including building construction, insulation, packaging, and the like. We provide an overview of granted patents (73) and pending applications (34) related to granted patents, the dominant patent portfolios (five, with the number of patents and/or applications per owner between six and 44), the patent owners, and highlight the key claims formulated to protect the inventions. Additionally, we outline various options towards an increased activity in the field.

**Conclusion:**

Patent developments in the field leave the impression that fungal materials, despite their high potential as renewable and biodegradable materials, have been held back due to patent over-protection. Considering the need for replacing current petroleum-based materials with renewable biomaterials, coordinated efforts may be called for to intensify efforts in the field.

**Supplementary Information:**

The online version contains supplementary material available at 10.1186/s40694-024-00177-2.

## Introduction

Fungal products contribute with more than 54 trillion US Dollar to the global economy [1]. This incredibly high value is generated in fungal biotechnology through harnessing the versatile metabolic power of several dozens of unicellular and filamentous fungi and the production of a very diverse set of products including food, beverages, pharmaceuticals, cosmetics, enzymes, and biofuels [1]. For comparison, the global market value of the food, chemical, automotive, and pharmaceutical industries was in 2022 8.75 trillion USD, 7.68 trillion USD, 2.56 trillion USD, and 1.28 trillion USD, respectively [2]. Fungal biotechnology is thus an important innovation driver and economic player.

Recent breakthroughs in fungal biotechnology could even potentiate its impact on human welfare and pave the way towards a new era in the materials and building sectors [3]. The 2021–2022 Special Issue ‘Connecting materials science with fungal biology’ published in this Journal (and the first of its kind in the scientific literature) brought together 12 publications that highlighted ‘breakthroughs in the fabrication of fungal mycelium-based materials, all of which have been made possible by the interdisciplinary and transdisciplinary collaboration of fungal biologists and biotechnologists with artists, designers, materials scientists, and architects.’ (In Ref. [4] and references therein). Many actors from academia, industry, art, design, and citizen science are thus engaged in the quest for new renewable materials based on fungal mycelium [5, 6] and market-driven research on fungal materials is conducted in several companies including MycoWorks (US), Ecovative Design (US), Mogu (IT), Loop Biotech (NL), Arup (UK), PLP labs (UK), GrownBio (NL), Mycotech lab (IDN), and Biotopa gGmbH (DE).

The innovation path towards exploiting fungal mycelium as building material, insulation material, or packaging aims to eliminate fossil dependency for material production in the built environment. The vision is to biotechnologically produce fungal mycelium-based materials, mainly composite materials, from renewable lignocellulosic agricultural and forestry resources [3]. In 2023, the Global Alliance for Buildings and Construction (GlobalABC) of the UN Environment Programme stated in their report ‘Building Materials and the Climate: Constructing a New Future’ that the ambitious target of net zero emissions from the built environment sector by mid-century could be achievable when about 40% of building materials are biobased and when the production of fungal mycelium-based building materials can be up-scaled [7]. The vision, the opportunities and challenges of a fungal biotechnology contributing to a sustainable construction industry are thus crystal clear. But how to get there?

It is generally accepted that patents benefit society because they push innovation and promote new services and products in that they set a basis for inventors to receive a revenue for their innovations, thereby stimulating industry and start-ups to innovate. As Abraham Lincoln so incisively put it: “The patent system adds the fuel of interest to the fire of genius” [8]. A patent is usually valid for 20 years from the filing date (not from the patent granting date) in the country or region where the application was filed. The patent gives its owner the right to exclude others from using, making, and/or selling a new technology or product that makes use of the invention as specified in the patent claims in the country where the patent is valid. The owner may permit others (with or without requesting reimbursement, e. g., through a license) to use the patent-protected invention. This is, however, not to be taken as a permission to manufacture a certain product that uses the invention, since additional permissions arising from other patents may be required. For example, a hypothetical first basic patent may claim a building block that includes mycelium, and a hypothetical second patent may improve the building block by additionally including a stabilizing rod. Neither the owner of the second patent nor a licensee of the second patent would be allowed to commercially use the building block that includes the stabilizing rod unless they also had permission from the owner of the first patent.

Notably, because patents are required to disclose the invention in a way that enables others to execute it, they also stimulate competitors and the public sector to innovate, in particular compared to a situation where a company treats the invention as a trade secret instead. Hence, there is a trade-off between disclosing an invention and the potential of obtaining a temporary protection for commercializing it [9]. Consequently, patent disclosures can be considered as important drivers of innovation because the inventions are freely accessible for everyone. Because of this, some companies deliberately disclose selected knowledge to the public domain to stimulate further innovations in the field [10]. Interestingly, there are also concerns of under-protecting and over-protecting intellectual property in emerging fields. Under-protection is considered as a lack of interest from companies and investors, over-protection hinders competition and may cause market barriers [11]. That said, what are the patenting trends for materials based on fungal mycelium? How vivid, innovative, and competitive is the field?

To answer these questions, we must go beyond what has been published thus far in the scientific literature regarding fungal mycelium-based patents. After the first patent survey that covered patent developments between 2009 and 2018 [12], more recent patent surveys addressed not only the materials field but also the overall intellectual property landscape of filamentous fungi [13–16]. We thus specifically summarize in this paper the current patent landscape of fungal mycelium-based materials for technical applications including building construction, thermal insulation, soundproofing, packaging, and the like. We provide an overview of both granted patents and pending applications related to (e. g., as family members of) granted patents, the dominant patent portfolios, the patent owners, and highlight the key claims formulated to protect the inventions.

## Results

### Patent and patent application search

The databases Espacenet of the European Patent Office and DEPATISnet of the German Patent and Trademark Office were used for the patent search. One of our goals was the identification of new application areas of fungal mycelium-based materials. Therefore, the patent search was not limited to specific patent classes. Likewise, no restrictions were made regarding country, patent office, and time window (e. g., filing date of patent application, publication date of patent application, granting date of the patent). In fact, it appears that the very first patent application in the field was filed in 2007 (US2013280791A1, US9803171B2), hence, all patents and patent applications listed currently in patent databases are still in force in 2024. A preliminary search limited to keywords “mycelium OR mycelial” together with “substrate OR carrier” in the summary was used for defining a set of parameters (Table 1) that keeps as many of the patents of interest as possible among the search results, while eliminating those patents from the results list that were directed towards other application fields, e.g. for food production.

**Table 1:** Search parameters for the patent survey

Occurring together in full text							
Mycelium OR mycelial	Substrate OR carrier	OR					Publication number includes B1 OR B2
		Composite NEAR* material	Mycelial NEAR composite	Mycelium NEAR scaffolding	Building NEAR material	Biodegradable NEAR material	

^*^: ‘NEAR’ was defined to mean three words or less apart from each other. Note that B1 and B2 refer to international patent nomenclature. B1: granted patent, B2: amended specification

Our first search was conducted in May 2023 and resulted in a list of 255 using Espacenet and 333 using DepatisNet for the same search parameters. The latter was expected to be more complete and was therefore used as a starting point that still included a reasonably large number of patents that were not of interest, for example patents regarding production of antibiotics or fungicides. Such patents were eliminated by hand (226 in total). The resulting final list thus included 107 patents, wherein EP patents are counted per country in which they are active, and in some cases pending family members. During our second search in May 2024, we were able to update this list for members of pending applications that had been granted in the meantime and some newly filed family members that had been published.

The final list covers a broad range of applications for fungal mycelium-based materials that are presently patent-protected or expected to be in the future. We also conducted a supplementary search for identifying patent applications that have no granted family members yet (for search parameters see Table 2).

**Table 2:** Search parameters for the supplementary survey

Occurring together in full text							
Mycelium OR mycelial	Substrate OR carrier	OR					Publication number includes NEITHER B1 NOR B2
		Composite NEAR* material	Mycelial NEAR composite	Mycelium NEAR scaffolding	Building NEAR material	Biodegradable NEAR material	

^*^: ‘NEAR’ was defined to mean three words or less apart from each other. Note that B1 and B2 refer to international patent nomenclature. B1: granted patent, B2: amended specification

We decided to group the search results into five different sub-groups according to the proposed technical application areas: (i) Building/Construction Materials, (ii) Textile Materials, (iii) Filtration Materials, (iv) Chitosan Materials, (v) Other Materials and Production Methods. We used the scheme shown in Table 3 for all five sub-groups (Tables 4, 5, 6, 7 and 8) to provide an overview on the main protected (or expected to be protected) subject matter (i.e. the most important claim), the inventors, countries, and the patent expiration date. For more detailed information, see Additional File 1, which also provides a link to the DEPATISnet document.

**Table 3:** Organization of entries in Tables 4, 5, 6, 7 and 8

Patent number	Publication date	Patent owner	Title	Country, patent expiration date
Additional patents or patent applications, expiration dates	Claim* (*optional comments by the authors of this study*)			

The first row and the “Claim” field refer to an index patent, whereas “Additional Patents or Patent Applications” lists family members (granted patents indicated by “B1”, “B2” or “C” at the end of the file number, and/or pending applications indicated by an “A” at or near the end of the file number) of the index patent. “Publication Date” is the publication date of the index patent (usually, the corresponding patent application was published earlier). “Country” refers to the jurisdiction where the index patent is in force, which is usually a country

^*^with key features highlighted in bold font

Table 3 summarizes properties of a few so-called “patent families”. Each patent family is based on at least one common priority application for a given invention, usually accompanied, or followed by applications in other countries and/or divisional applications to one or more of those applications. Often, the technical content of the description remains essentially the same across the family members, but the claims that define the scope of the protection may differ for different jurisdictions. Note that an EP patent (patent number starting with “EP”) is granted for a bundle of countries, but whether the patent is in force in a given country depends on further conditions, for example payment of annual fees, filing of translations, etc. For EP patents, countries in which the patent was abandoned are not listed. A PCT application (publication number starting with WO) refers to an application filed with the Patent Corporation Treaty (PCT) which is relevant for 157 PCT contracting states. Note that there is no such thing like a “PCT world patent”. For each country or region (like EP) where protection is sought, an individual patent needs to be obtained, initiated by entering the national or regional phase of the PCT application within a specified period. A PCT application may be described as a “foot in the door” that allows to postpone the decision of whether or not to seek a patent for a certain jurisdiction. Listed in Tables 4, 5, 6, 7 and 8 are only PCT applications where the time limit for entering the national/regional phase has not yet expired. The “Claim” field reproduces, for information purposes, one of the independent claims of the index patent. Even though a patent is supposed to be directed at a single invention, the index patent may include further independent claims directed at other aspects of the invention, and claims of the family members may usually differ somewhat from the claim(s) of the index patent. See Additional File 1 for more information on the family members.

The information provided in Table 9 summarizes current patent applications (status May 2023 with an update from May 2024 for the respective family members). Its organization differs slightly from that of Tables 4, 5, 6, 7 and 8: The claim provided in the second row refers to claim 1 as originally filed, and instead of the (not yet existing) patent expiry date, the link to the original document is provided in the last column of the first row.

### Geographical distribution of patents and patent owners

**Fig. 1:**
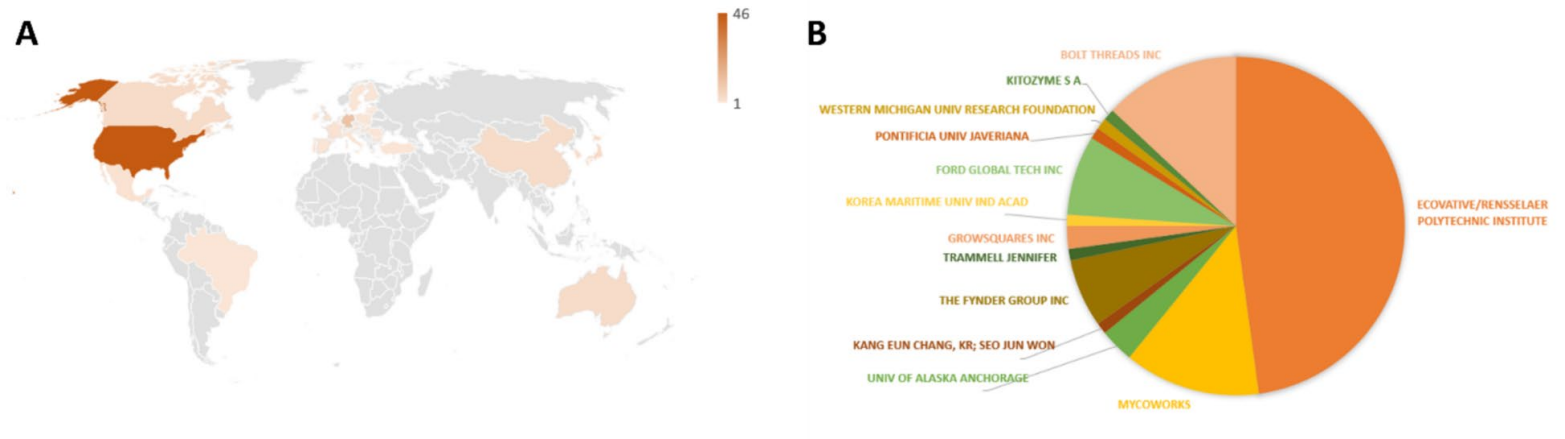
Patenting countries (**A**) and patent owners (**B**) of issuance for materials based on fungal mycelium

Figure 1A illustrates the regional distribution of the above listed patents and their pending family members (excluding the four EP applications—EP3599832A4, EP3484995A4, EP3860370A4, EP3973055A4—originating from PCT applications, for which it is not yet clear if and for which EP countries they may eventually be relevant). Striking is the large dominance of the US in terms of granted/pending patents (50), while most countries that are represented in Fig. 1 have only one patent that is presently in force or pending. However, Japan has five, China, Australia, and Canada have four each, South Korea and Israel have three each, and eight countries have two patents each in force or pending. Remarkably, about two thirds (70/114) of the patents that are currently in force (and their pending family members) are held by three owners: Ecovative (some together with Rensselaer Polytechnic Institute), Mycoworks, and Bolt Threads Inc (Fig. 1B).

**Table 4:** Building/Construction Materials

**US11293005B2**	**05.04.2022**	**ECOVATIVE DESIGN LLC, US**	**Process for making mineralized mycelium scaffolding and product made thereby**	**US** **19.08.2039**
No other countries	A structure comprising: • A scaffold of fungal biopolymer of predetermined form characterized in being formed of a network of interconnected mycelia cells; and • A coating of at least one of an apatite, a carbonate, and a silicate on at least some of said cells within said network			
**US10604734B2**	**31.03.2020**	**UNIV OF ALASKA ANCHO-RAGE, US**	*Thermal insulation material from mycelium and forestry byproducts*	**US** **27.01.2037**
CN108699507A, US20200255794A1	A biodegradable insulation material comprising: • A structural scaffold comprising a three-dimensional structure and a mycelium from a first temperature resilient fungus, wherein the mycelium from the first temperature resilient fungus has colonized the three-dimensional structure, wherein the structural scaffold has a chitinous hydrophobic outer skin; and • A substrate comprising nutritive media and a mycelium from a second temperature resilient fungus, wherein the mycelium from the second temperature resilient fungus has colonized the substrate; • Wherein the biodegradable insulation material is the result of the structural scaffold and substrate fusing together, • Wherein the first temperature resilient fungus and second temperature resilient fungus are different			
**KR101933573B1**	**28.12.2018**	**KANG EUN CHANG, KR; SEO JUN WON, KR**	**Method for manufacturing a human-friendly functional panels using biopolymers**	**KR** **22.10.2038**
No other countries	In a method of manufacturing a functional plate material using discarded or unused biomass resources and mushroom mycelium, a biopolymer, a sterilized biomass acquisition step of collecting biomass and sterilizing it; and on a sterilized PDA medium. Cultivating biopolymer spores based on basidiomycetes; Collecting a portion of the cultured biopolymer spores to prepare a liquid biopolymer substrate or a solid biopolymer substrate; The sterilized biomass and biopolymer A first mixture acquisition step of obtaining the first mixture by adding and mixing the base material, nutrients, and moisture; A first growth step of adding the first mixture into a cave mold and growing it; Removing the mold and forming the first mixture; and a second growth step of growing the primary growth into a second growth (*It is not quite clear, which fungal species (if any) is supposed to be used, since it is referred to as ‘biopolymer mushroom’*)			
**!!!** **US9410116B2**	**09.08.2016**	**MYCOWORKS INC, US; ROSS PHILIP, US**	**Method for producing fungus structures**	**US** **23.09.2032**
US9951307B2 (18.12.2031); CN103547668A; US10947496B2 (28.11.2031); US2021198621A1	1. A method for growing organically derived building material in the form of a moldable substrate to serve a wide range of manufacturing and construction applications, the method comprising the steps of: a) Obtaining a lignocellulose based medium capable of supporting the growth of saprophytic fungi; b) Mixing said lignocellulose based medium with water to reach a hydration level; c) Inoculating said lignocellulose based medium with a fungal inoculum; d) Allowing time for said inoculated lignocellulose based medium to become colonized to the extent that said inoculated lignocellulose based medium is transformed into a fungal mycelium without any secondary organisms displacing the process through infection; e) Providing a vessel in which said allowing step occurs and wherein environmental conditions in said vessel are regulated; f) Placing said fungal mycelium into a mold such that the fungal mycelium forms into a fungal molded shape; g) Applying a primary compressive pressure of at least 100 PSI to the lignocellulose based medium, wherein before applying the primary compressive pressure, a plurality of rods and a plurality of slats of organic material-are layered near the top and the bottom of surface substrate, where each of the plurality of rods and the plurality of slats of organic material—and are positioned at right angle to each other to increase the structural capacities of the organically derived material; h) Reducing said primary compressive pressure by a factor of at least 4; i) Removing said rod and slat embedded fungal molded shape from said mold; and j) Drying said fungal molded shape at a specific temperature for a specific time period			
**!!!** **EP2702137B1**	**05.03.2014**	**ECOVATIVE DESIGN LLC, US**	Method for making dehydrated mycelium elements and product made thereby	**DE, NL, SE, DK, FR, PL, GB** **25.04.2032**
JP5922225B2; CA2834095C; AU2012249802B2, all 25.04.2032	A method of making dehydrated mycelium elements comprising the steps of: 1) Creating a living hydrated mycelium composite containing at least one of a combination of mycelium and fibers, mycelium and particles, and mycelium, particles and fibers; • Adding a nutrient material to said mycelium composite in an amount to promote mycelia tissue growth; • Thereafter dehydrating the mycelium composite to a moisture content of less than 50% by weight to deactivate the further growth of mycelia tissue to form a dehydrated mycelium element; • Thereafter storing the dehydrated mycelium element at a temperature in the range of from -50 °F to + 200 ºF (-45.56 to 93.33 ºC); and • Adding moisture to said mycelium element in an amount sufficient to rehydrate said mycelium element and to re-activate mycelium on the exterior of said mycelium for growth into an adjacent mycelium element to bond said elements together to form a fabricated section; OR 2) Creating a living hydrated mycelium composite containing at least one of a combination of mycelium and fibers, mycelium and particles, and mycelium, particles and fibers; • Adding a nutrient material to said mycelium composite in an amount to promote mycelia tissue growth; • Thereafter dehydrating the mycelium composite to a moisture content of less than 50% by weight to deactivate the further growth of mycelia tissue; • Processing the dehydrated mycelium composite into a plurality of discrete particles; and • Adding moisture to said plurality of discrete particles in an amount sufficient to re-hydrate said discrete particles and to re-activate mycelium on the exterior of said discrete particles for growth into adjacent discrete particles			

Patents having an unfavourable broad scope of protection are marked in the Patent Number field with !!!

**Table 5:** Textile Materials

**US11118305B2**	**14.09.2021**	**THE FYNDER GROUP INC, US**	**Fungal textile materials and leather analogs**	**US 17.06.2040**
CN114901902A; US11414815B2 (17.06.2040); US11427957B2 (17.06.2040); US11447913B2 (17.06.2040); US11643772B2 (17.06.2040); US11649586B2 (21.07.2041) US11718954B2 (17.06.2040); US11952713B2 (17.06.2040); KR20220024666A; JP2022538816A; CA 3143603A1; TW202116537A; EP3986186A4	A method for making a durable sheet material, comprising: a) Contacting an inactivated fungal biomass with an aqueous solution comprising a liming substance; b) Contacting the inactivated fungal biomass from step (a) with an aqueous solution comprising a deliming substance; c) Contacting the inactivated fungal biomass from step (b) with an aqueous solution comprising a polymer; d) Contacting the inactivated fungal biomass from step I with an aqueous solution comprising a crosslinker; e) Contacting the inactivated fungal biomass from step (d) with an aqueous solution comprising a plasticizer; f) Drying the inactivated fungal biomass from stI(e) to form a dried inactivated fungal biomass; and g) Heat-pressing the dried inactivated fungal biomass to form the durable sheet material (*the various US patents vary in specifics of the method, e. g. by specifying a ratio of fungal biomass:polymer, or by providing very detailed process instructions*)			

Textile materials with their inherent sheet-shaped structure can also be produced from sheet-shaped or dissolved mycelium, in other words, without a substrate or carrier. These are therefore not included in our survey. Some examples include CN113501994B, DK181371B1, KR102536510B1, and NL2026370B1

**Table 6:** Filtration Materials

**US10087094B2**	**02.10.2018**	**PONTIFICIA UNIV JAVERIANA, CO**	**Consortium of fungi immobilized on a laminar lignocellulose carrier for the treatment of wastewater and method for producing same**	**US 09.12.2034**
	A laminar biocarrier for the treatment of wastewaters with an elastic, flexible and resistant mesh shape, wherein said laminar biocarrier is prepared by weaving or interlacing lignocellulosic yarns, and holds and immobilizes a wood-decay fungi biomass layer			
**US9714180B2**	**25.07.2017**	**BAYER EBEN, US; ECOVATIVE DESIGN LLC, US; MCINTYRE GAVIN, US; SCULLY CHRISTOPHER, US**	**Composite material for absorbing and remediating contaminants and method of making same**	**US 12.01.2034**
	A composite material comprising a mass of pellets, each said pellet composed of a saprophytic fungi strain characterized in producing an enzyme capable of breaking down animal waste and a particulate material wherein said fungi forms a plurality of hyphae bonded to said particulate material

**Table 7:** Chitosan Materials

**US9982393B2**	**29.05.2018**	**WESTERN MICHIGAN UNIV RESEARCH FOUNDATION, US**	**Chitosan as a biobased barrier coating for functional paperboard products**	**US 28.11.2036**
	A composite fiber stock material comprising: at least one layer of a fibrous base sheet; and at least one chitosan layer comprising a chitosan coat weight from about 1 g/m2 to about 10 g/m2; • Wherein the composite fiber stock material has an air permeance from about 20 nm/Pa s to about 50 nm/Pa s • Aerating the aqueous solution after a suitable exposure time to the chlorine dioxide, • Wherein the aerating halts the adverse effect of the chlorine dioxide on the microorganism biomass autolysate; and • Providing a volume of an organic solvent to float on top of the aqueous solution, after said treating, wherein the volume of the organic solvent is sufficient to dissolve the lipids of the autolysate; • Wherein said providing the volume of the organic solvent causes the autolysate to separate into an upper layer comprising the organic solvent and the lipids; and a lower layer comprising the aqueous solution and non-lipid materials of the autolysate; • Wherein said upper layer protects said lower layer from environmental microbial contamination (*The chitosan is obtained from fungi: The patent discloses ‘methods of deriving unique chitosan compositions from chitin and chitosan-containing fungal biomass’*)

**Table 8:** Other Materials und Production Methods

**US11638671B2**	**02.05.2023**	**TRAMMELL JENNIFER, US**	**Mycelium composite burial container**	**US 10.09.2041**
	A container for holding a deceased or their remains comprising: a) An outer surface made at least in part of mycelium composite, wherein the outer surface comprises a plurality of equally spaced apart grooves on at least a majority of the outer surface; b) An opening for receiving the deceased or their remains; c) A lid made at least in part of mycelium composite, wherein the lid is configured to completely seal the opening; and d) One or more pegs made at least in part of mycelium composite, each peg comprising a first end and a second end distal from the first end, the first end configured for insertion into any of the plurality of grooves, the second end consisting essentially of a decorative element			
**US11359074B2**	**14.06.2022**	**ECOVATIVE DESIGN LLC, US**	**Solution based post-processing methods for mycological biopolymer material and mycological product made thereby**	**US** **12.08.2038**
JP7161489B2 (29.03.2038); BR112019020132B1 (29.03.2038); AU2018243372A1 (pending); CA3058212A1 (pending); EP3599832A4 (pending); CN110506104B (29.03.2038); US20230013465A1 (pending)	A method comprising the steps of: • Obtaining a tissue consisting essentially of fungal mycelium containing native moisture, wherein said fungal mycelium is free of any stripe, cap or spores; • Treating said tissue with an organic solvent solution for a period of time sufficient to permit permeability into the tissue while desiccating the tissue to replace said native moisture with said solvent solution; • Removing said tissue from said solution; • Pressing said tissue to a minor thickness thereof; and • Thereafter drying said tissue, thereby providing a processed mycological biopolymer having a density within a range of 15 pcf to 50 pcf			
**US11310968B2**	**26.04.2022**	**MYCOWORKS INC, US**	**System for growing fungal materials**	**US** **14.07.2037**
US10842089B2 (21.11.2038); US10687482B2 (27.06.2038); US11013189B2 (14.07.20238); US11793124B2 (14.07.2023); EP484995A4; MX2019386A (pending)	A scaffold structure for growing fungi comprising: a) A nutrient substrate comprising evenly distributed fungal inoculum; b) A porous material positioned away from said nutrient substrate and defining an intermediate layer which does not readily bind with fungal tissue and provides uniform initial conditions of growth, wherein the porous material is microperforated or woven and selected from the group consisting of metal, plastic, and ceramic plate; c) A closed administrable space positioned away from said nutrient substrate and said porous material; d) A first layer of fungal tissue connecting said nutrient substrate to and through said porous material to said administrable space; e) A successive layer of fungal tissue within said administrable space; f) A growth field comprising growth field locations such that growth of said first layer of fungal tissue is directed through said growth field locations so as to create said successive layer of fungal tissue comprising fungal hyphae; and g) A portion of fungal material delaminated from said intermediate layer, the delaminated portion being different from said fungal tissue in that the delaminated portion is chemically or physically altered			
**US11359174B2**	**14.06.2022**	**ECOVATIVE DESIGN LLC, US**	**Bioreactor paradigm for the production of secondary extra-particle hyphal matrices**	**US** **01.05.2040**
US20230056666A1; AU2019352842A1; CA3113935A1; EP3860370A4; all pending	A method of producing a mycological material comprising the steps of providing a vessel having a chamber; loading a substrate of discrete elements inoculated with a filamentous fungus into said chamber; feeding a pre-conditioned air stream through said vessel for diffusion between said discrete elements in said chamber and for a time sufficient for said filamentous fungus to expand a contiguous network of hyphae between and around said discrete elements to form an isotropic inter-particle hyphal matrix; and continuing to feed said pre-conditioned air stream through said vessel for diffusion between said discrete elements and said isotropic inter-particle hyphal matrix for a time sufficient to develop a polarized condition within said vessel wherein air exits said isotropic inter-particle hyphal matrix as a laminar flow into at least one void space within said vessel and to form an extra-particle hyphal matrix extending from said isotropic inter-particle hyphal matrix in the direction of airflow within said at least void space			
**!!!** **US11277979B2**	**22.03.2022**	**ECOVATIVE DESIGN LLC, US**	**Mycological biopolymers grown in void space tooling**	**US** **15.06.2036**
US20220290199A1 pending	A process of growing a mycological biopolymer material, comprising the steps of a) Providing a tool defining a cavity therein with an opening into said cavity; b) Packing said cavity of the tool with nutritive substrate and a fungus; c) Placing a lid on said tool to cover said cavity, said lid having only one outlet therein defining a void space open to fresh air; d) Allowing said fungus to grow mycelium within said cavity and to allow the mycelium to respirate within said cavity thereby producing carbon dioxide while colonizing the nutritive substrate; e) Allowing the produced carbon dioxide to diffuse out of said outlet in said lid to create a gradient of carbon dioxide; and f) Allowing the mycelium to grow along said gradient to fill said void space without producing a stipe, cap or spore therein and to produce a mycelium biopolymer in said void space (*even though the features sound carefully phrased, it appears as if the subject matter was realized by a substrate with mycelium for food mushroom production that is arranged in a bucket with a partially closing lid, see Discussion*)			
**US10945382B2**	**16.03.2021**	**GROWSQUARES INC, US**	**Soil module and method of manufacture thereof**	**US** **07.11.2038**
US11102938B2 (07.11.2038)	A self-contained soil module, the self-contained soil module comprising: • A biodegradable outer frame forming an outer surface of the self-contained soil module comprising a top side, a bottom side, and one or more side walls, wherein the biodegradable outer frame is constructed from at least a mycelium sheet, the mycelium sheet grown from a mycelium substrate mixture, the mycelium substrate mixture configured to grow such that the mycelium substrate mixture fully forms at least one of the top side, the bottom side, and the one or more side walls; • A soil composition disposed within the biodegradable outer frame; and • An inner layer comprising a biodegradable wrapping disposed within the biodegradable outer frame, the biodegradable wrapping comprising an upper sheet and a lower sheet, wherein the upper sheet and the lower sheet are situated in parallel planes; • At least one plant seed of at least one type of plant disposed between the upper sheet and the lower sheet; and • A grid structure disposed on the bottom side, the grid structure in contact with the one or more side walls			
**US11015059B2**	**25.05.2021**	**BOLT THREADS INC, US**	**Composite material, and methods for production thereof**	**US** **22.05.2040**
US11891514B2 (22.05.2040); SG11202112275VA; CA3137693A1; MX2021014233A; TW202112943A; CN114127278A; JP2022534025A; AU2020279832A1; EP3973055A4; KR20220027075A	A composite mycelium material, comprising: • A cultivated mycelium material comprising one or more masses of branching hyphae, wherein the one or more masses of branching hyphae is disrupted; and • A bonding agent selected from the group consisting of a vinyl acetate-ethylene (VAE) copolymer, a vinyl acetate-acrylic copolymer, a polyamide-epichlorohydrin resin (PAE), a copolymer, transglutaminase, citric acid, genipin, alginateegotiarabic, latex, a natural adhesive, and a synthetic adhesive			
**!!!** **EP2094856B1**	**08.11.2016**	**ECOVATIVE DESIGN LLC, US**	**Method for producing grown materials and products made thereby**	**13.12.2027** **ES, PL, SI, CH, HU, BE, TR, SK, SE, RO, PT, NL, LV, LT, IT, IE, GB, FR, FI, DK, DE, BG, AT**
US9485917B2 (31.07.2035); US8999687B2 (18.05.2028); US10525662B2 (27.03.2028); US9801345B2 (31.03.2030); US9795088B2 (03.02.2029; US10589489B2 (11.10.2028); US10583626B2 (28.08.2028); US11932584B2 (21.05.2028); JP5740492B2, JP5457194B2 (both 13.12.2027); CA 2672312C (13.12.2027); NZ 578415A (pending); CN 101627127B (13.12.2027); AU2007333545B2 (13.12.2027); IL199315A (Filing Date + 20 years: 11.06.2029); IL234585A, IL234584B (both Filing Date + 20 years: 11.09.2034)	A method of making a composite material characterized in the steps of forming an inoculum including a preselected fungus; forming a mixture of a substrate of discrete particles and a nutrient material, said nutrient material being capable of being digested by said fungi; adding said inoculum to said mixture; and Allowing said fungus to digest said nutrient material in said mixture over a period sufficient to grow hyphae and to allow said hyphae to form a network of interconnected mycelia cells through and around said discrete particles thereby bonding said discrete particles together to form a self-supporting composite material (*Opposition had been filed on December 29, 2016, by CNC Holding but was withdrawn later, despite the preliminary opinion being in favour of the opponent.*)			
**KR102001771B1**	**18.07.2019**	**KOREA MARITIME UNIV IND ACAD, KR**	**Manufacturing method for eco-friendly working materials with coffee waste**	**KR** **12.02.2038**
none	A method combining coffee grounds, sawdust and *Pleurotus eryngii*			
**US08313939B2**	**20.11.2012**	**FORD GLOBAL TECH INC, US; JOHNSTON et al., US**	**Injection molded mycelium and method**	**US** **29.12.2030**
none	A method of making a molded part, comprising: • Forming a mixture of a fungal inoculum, a nutrient source for the fungal inoculum, and a liquid; • Injecting the mixture into a first mold cavity; • Sealing the first mold cavity against a second mold cavity; • Growing live mycelium from the fungal inoculum to fill the first and second mold cavities to form a first molded part; • Curing the live mycelium to terminate further growth; • Separating the first mold cavity and the second mold cavity; • Injecting a mycelium slurry over the first molded part in the first mold cavity; • Sealing the first mold cavity against a third mold cavity; • Growing live mycelium from the mycelium slurry to form a second molded part over the first molded part; and • Curing the live mycelium of the second molded part to terminate further growth and develop a dual mycelium molded part made up of the first molded part and the second molded part			
**US8298809B2**	**30.10.2012**	**FORD GLOBAL TECH LLC, US; EDWARD, US; ALAN, US**	**Method of making a hardened elongate structure from mycelium**	**US** **13.08.2030**
none	A method of making a hardened elongate structure, comprising: • Growing mycelium to produce a live mycelium mat having a thickness between approximately 0.125 inches (0.3175 cm) and 2.0 inches (5.08 cm) and having branching hyphae; • Layering the live mycelium mat to form an elongate multi-layered structure; • Allowing the hyphae to grow inward into the multi-layered structure such that the hyphae are interwoven throughout the multi-layered structure; and • Curing the multi-layered structure by heating the structure to a temperature of at least 150 degrees Fahrenheit for a period of at least one day to terminate mycelium growth and form a hardened elongate structure			
**US08298810B2**	**30.10.2012**	**FORD GLOBAL TECH LLC, US; EDWARD, US; ALAN, US**	**Mycelium structure with self-attaching coverstock and method**	**US** **25.12.2030**
none	A method of making an injection molded part, comprising: • Combining a fungal inoculum with a liquid and a nutrient source to form a mixture; • Inserting a coverstock into a hydraulic press injection mold having a closed mold cavity; • Injecting the mixture into the closed mold cavity of the hydraulic press injection mold through an injection port; • Growing live mycelium from the mixture that fills the closed mold cavity and physically couples with the coverstock; and • Heating the live mycelium to terminate further growth and develop an injection molded part made of mycelium and the coverstock for use in a vehicle interior			
**US08283153B2**	**09.10.2012**	**FORD GLOBAL TECH LLC, US; EDWARD, US; ALAN, US**	**Mycelium structures containing nanocomposite materials and method**	**US** **09.06.2030**
none	A method of making a molded part, comprising: • Mixing an aggregate with a fungal inoculum to form a mixture; • Evenly distributing nanoparticles throughout the mixture; • Inserting the mixture into a mold cavity; • Growing live mycelium to fill the mold cavity; and • Curing the live mycelium to terminate further growth of the molded part			
**US08227224B2**	**24.07.2012**	**FORD GLOBAL TECH LLC, US; EDWARD, US; ALAN, US**	**Method of making molded part comprising mycelium coupled to mechanical device**	**US** **04.09.2030**
none	A method of making a molded part, comprising: • Inserting a fungal inoculum and a mixture comprising a liquid and a nutrient for the fungal inoculum into a mold cavity; • Inserting a portion of a mechanical device into the mold cavity such that a portion of the mechanical device is exposed by not being inserted in the mold cavity; • Growing the fungal inoculum into live mycelium that operably couples with the portion of the mechanical device inserted in the mold cavity, and such that the mycelium does not couple to exposed portion of the mechanical device; and • Heating the mycelium to terminate further growth and develop a molded part made of mycelium and the mechanical device			
**US08227225B2**	**24.07.2012**	**FORD GLOBAL TECH LLC, US; EDWARD, US; ALAN, US**	**Plasticized mycelium composite and method**	**US** **01.09.2030**
none	A method of making a mycelium structure, comprising: • Dissolving a soluble plastic film having insoluble polymer particles in a liquid to form a solution of polymer particles; • Combining the solution of polymer particles with a fungal inoculum and a nutrient source for the inoculum to form a mixture; • Growing a live mycelium network that bonds with the polymer particles to form a plasticized structure; and • Terminating growth			
**US08227233B2**	**24.07.2012**	**FORD GLOBAL TECH LLC, US; EDWARD, US; ALAN, US**	**Method of making foamed mycelium structure**	**01.09.2030**
None	A method of making a foamed mycelium structure, comprising: • Providing a fungal inoculum having a fungus capable of growing hyphae; • Adding the fungal inoculum to a liquid and a nutrient source for the inoculum to form a slurry; • Placing the slurry in a reaction vessel having an agitation device; • Agitating the slurry in the presence of at least one select gas to create gas bubbles in the slurry; • Allowing the fungus to grow a live mycelium network through and around the gas bubbles; and • Terminating growth			

Patents having an unfavourable broad scope of protection are marked in the Patent Number field with !!!

**Table 9:** Current patent applications

**US20230114815A1**	**13.04.2023**	**FS INSULATION B V, NL**	**Method of manufacturing a prefab construction element**	**US'815**
AU2021234132A1; CA3171060A1; EP3878943A1; EP4118186A1, WO2021180948A1 (past deadline for entering national/regional phase)	A method of manufacturing a prefab construction element for frame construction, comprising the steps of: • Providing a roofing, flooring or wall panel, which panel comprises an enclosure, • Providing at least one fungus and a substrate, • Introducing or preparing a mixture of the at least one fungus and the substrate, in the enclosure and • Allowing the at least one fungus to grow to form a network of hyphae through the mixture and into the walls of the enclosure to form a mycelium composite, and • Drying the composite while it remains in the enclosure of the panel			
**WO2022155516A1**	**21.07.2022**	**MASSACHUSETTS INST TECHNOLOGY, US; STANDARD BANK GROUP LTD, ZA**	**Method for mycotecture**	**WO'516**
US2022217923A1 (past deadline for entering national/regional phase)	A method of making a food source or medicinal composition and of making a mycotecture construction unit, comprising: (a) Cultivating a mycelium/substrate composite and edible mushrooms, including; (i) Forming a mycelium inoculum of a preselected fungus; (ii) Inoculating a substrate with the mycelium inoculum; (iii) Growing mycelium in the substrate from the mycelium inoculum; and (iv) Harvesting the edible mushrooms from the mycelium, leaving a mycelium-substrate composite; (b) And manufacturing the mycotecture construction unit, including; (i) Placing the mycelium-substrate composite into a press having a cavity with a predetermined shape; (ii) Pressing the mycelium-substrate composite at a predetermined pressure; and (iii) Heating the mycelium-substrate composite *(Dual use of harvesting edible mushrooms and using the substrate for construction purposes)*			
**WO2022068092A1**	**07.04.2022**	**TIANJIN INST OF INDUSTRIAL BIOTECHNOLOGY CHINESE ACADEMY OF SCIENCE, CN**	**Use of mycelium material in oil absorption**	**WO'092**
CN112225326A (past deadline for entering national/regional phase)	Use of a mycelial material for oil absorption, wherein the mycelial material consists of fungal hyphae (*This claim appears to be incredibly broad)*			
**US20220073865A1**	**10.03.2022**	**RISE INNVENTIA AB, SE; TECHNION RES & DEV FOUNDATION, IL**	Mycelium-containing hybrid materials	**US'865**
none	A method of preparing a composition comprising mycelium of a fungus and a cellulose, the method comprising inoculating a liquid medium with said fungus, said liquid medium comprising nutrients and said cellulose, thereby obtaining said composition comprising said mycelium and said cellulose			
**US20210403857A1**	**30.12.2021**	**ISA TANTEC LTD, CN**	Method of producing mycelium textile fabric and fabrics and products made thereby	**US'857**
WO2021259378A1 (past deadline for entering national/regional phase)	A system for creating mycelium fabric products, comprising: a bioreactor configured to grow mycelium; substrate or feeder materials provided in the bioreactor; a single batch reactor configured to receive grown mycelium panels; treatment solutions provided in the single batch reactor to treat the mycelium panels and create treated mycelium panels; a temperature controlled vacuum oven configured to receive treated mycelium panels; and a cutting assembly			
**EP3828260A1**	**02.06.2021**	**UNIV CATOLICA PORTUGUESA UCP, PT**	**Composite biomaterial, obtaining methods and uses thereof**	**EP'260**
none	A composite biomaterial comprising: a woody material as a support; a substrate selected from the following list: seed, cereal, woody material or combinations thereof; a mycelium as an aggregating agent; wherein the woody material is simultaneously support, substrate and inoculum; wherein the mycelium growth aggregates the several components; wherein the mycelium belongs to a species selected from the following list: *Pleurotus ostreatus, Trametes versicolor, Ganoderma lucidum, Fistulina hepatica, Lentinus edodes; preferably Pleurotus ostreatus*			

## Discussion

While some of the patents that are currently in force in the field of materials based on fungal mycelium protect a highly specific subject matter that may be relevant only in the context of certain specialized production processes, a few have a very broad scope of protection. This, however, in our view in our view limits new commercial activities in the field due to over-protection. We will therefore discuss in the following such patents and the concerns related. We will refer to previous inventions from the 1940ies – 1950ies and to tacit knowledge from decades of experience in mushroom agricultural cultivation that, in our opinion, were neglected or ignored when the respective patents were granted*.*

### EP2094856B1 (EP856, see Table 8)

Granted claim 1 specifies the following:

A—A method of making a composite material characterized in the steps of

B—forming an inoculum including a preselected fungus;

C—forming a mixture of a substrate of discrete particles and a nutrient material, said nutrient material being capable of being digested by said fungi;

D—adding said inoculum to said mixture; and

E—allowing said fungus to digest said nutrient material in said mixture over a period sufficient to grow hyphae and

F—to allow said hyphae to form a network of interconnected mycelia cells through and around said discrete particles

G—thereby bonding said discrete particles together to form a self-supporting composite material.

This succession of methodical steps is clearly so basic that it contains no specific technical knowledge, application, or mycological skillset. Indeed, these steps are mandatory when a mycelium is to be grown on/in a substrate that includes discrete particles. In fact, the pioneering work of the German mycologist and engineer Walther Luthardt in the 1940s – 1950s laid the technical foundations for controlled cultivation of fungal mycelium on lignocellulosic substrates such as wood and sawdust. Therefore, controlled solid-state fermentation and thus fungal composite production with filamentous fungi is a technology that is at least 80 years old [17]. Arguably, the technology may even be older, but this is the time frame for which easily accessible publications exist. Luthardt’s very first patents from 1944 (DD000000000292B1) and 1951 (DD000000003114A1) describing these inventions and the underlying cultivation steps have been highlighted in [17] and honoured in 1964 with the short film *Mykoholz* (*Mycowood*) [18]. This 13 min film clearly demonstrates that there was already an industry in the former German Democratic Republic that produced mycelium-based materials harnessing the methodological steps as described above in claim 1 of EP2094856B1 (EP856). We thus argue that the latter does not include any unique, specific feature or step that may usually not be present, unless the composite material of EP856 is to be produced. In other words, virtually every use of composite mycelium materials – including preparations for growing edible mushrooms—may possibly fall under the scope of protection of EP856 and some of its family members (JP5457194B2, US10589489B2, US10583626B2). It should be noted that the cultivation and manufacturing of edible mushrooms have been well and in detail described in the mushroom agriculture literature from the 1950s to 1960s. For an example, the reader is referred to the book ‘*Die Champignonkultur, Grundlagen und Fortschritte im Garten- und Weinbau* ‘ (‘*Mushroom cultivation, basics and progress in horticulture and viticulture*’) published in 1958 [19].

Since the scope of protection varies to some degree between the family members, Additional File 2 gives an overview regarding features of the family member patents of EP856. In this context it is interesting to note that opposition was filed with the European Patent Office (EPO) against EP856 on December 29, 2016, by CNC Holding. In the preliminary opinion, the opposition division concluded that the claims 1 to 7, 13 to 15, 17, 18, 26, and 27 lack novelty over at least one of the following three prior art publications (and claim 1 over all of them): US5074959A; Rush Wayne: ‘Growing Mushrooms the Easy Way—Home Mushroom Cultivation with Hydrogen Peroxide—Volume I’ from 2001 [20]; and Philip Ross: “PURE CULTURE 1997-present” from 2016 [21]. However, the opposition was withdrawn on April 3, 2018, and the opposition division announced on April 30, 2018, that opposition proceedings are discontinued, and the patent was maintained as granted. Given the preliminary opinion of the EPO board of opposition, chances may be reasonable that a nullity suit against any patent that has the scope of protection of EP856 may result in the respective patent being revoked. However, an opt-out has been filed for EP856 regarding the Unified Patent Court (UPC), which means that it is not possible to file a single nullity suit with the UPC. Instead, nullity suits would have to be filed individually with local courts in each country where the patent is to be challenged. Very (or perhaps not very) surprising, Ecovative Design LLC (“Ecovative”) announced on September 15, 2023, ‘the Ecovative European Open Patent Program for Composite Materials to achieve the following goals with respect to composites that include mycelium materials’ to ‘Unleash Mycelium’s Potential together’, to ‘Collaborate with Integrity’, and to ‘Foster Innovation and Protect Ideas’ [22]. In brief, Ecovative announces that patent EP2094856B1 will not be enforced in the EU, and neither its Israeli counterpart in Israel if the technology is used without a licence. It indicates free(er) use of this technology, as Ecovative agrees to refrain from enforcing the rights arising from the European patent EP2094856B1 and the Israeli patent IL199315 against other participants. But in return the Program demands that the participants refrain from taking action against these patents on the one hand, and from enforcing their rights arising from further developments against Ecovative and Ecovative's cooperation partners on the other hand, i.e. to make these freely available to Ecovative until the further development patent expires. As we understand it, ‘further development’ here means that it is a patent that would actually have required permission from Ecovative to be used on the basis of the aforementioned EP or IL patent. (This is because a patent gives the owner the possibility to prohibit others from using/marketing the protected object, but not automatically the permission to use/market the protected object oneself, because this often requires a permission/licence from the owner of the basic patent.) However, a prohibition on filing one's own patent applications or on marketing them to others is not meant by that. A peace offer to re-stimulate competition, which has been limited due to Ecovative’s own patent over-protection? In that case, it may be surprising that the Program is limited to the EP and Israeli patents, rather than including, for example, at least those family members that have a similarly broad scope of protection (e. g., some US, CA, CN, and JP, see Additional File 2).

### US11277979B2 (US979, see Table 8)

Granted claim 1 specifies the following:

A—A process of growing a mycological biopolymer material, comprising the steps of.

B—providing a tool defining a cavity therein with an opening into said cavity;

C—packing said cavity of the tool with nutritive substrate and a fungus;

D—placing a lid on said tool to cover said cavity, said lid having only one outlet therein defining a void space open to fresh air;

E—allowing said fungus to grow mycelium within said cavity and to allow the mycelium to respirate within said cavity thereby producing carbon dioxide while colonizing the nutritive substrate;

F—allowing the produced carbon dioxide to diffuse out of said outlet in said lid to create a gradient of carbon dioxide; and

G—allowing the mycelium to grow along said gradient to fill said void space without producing a stipe, cap or spore therein and to produce a mycelium biopolymer in said void space.

Also for this patent, we argue that very fundamental tacit knowledge of mushroom agriculture industry has been claimed. For example, for growing edible mushrooms by techniques known for decades, a mycelium-inoculated substrate is placed in a container with a lid that has usually access to fresh air. In our opinion, the patent thus lacks novelty but limits competition unjustifiably and thus poses further market barriers to other actors in the materials science field exploiting fungal mycelium as a renewable resource.

### US9951307B2 (US307, see Table 4)

Granted claim 1 specifies the following:

A—A method for growing organically derived building materials in the form of a moldable substrate to serve a wide range of manufacturing and construction applications, the method comprising the steps of:

B—obtaining a lignocellulose based medium capable of supporting the growth of saprophytic fungi;

C—mixing said lignocellulose based medium with water to reach a hydration level;

D—inoculating said lignocellulose based medium with a fungal inoculum;

E—allowing time for said inoculated lignocellulose based medium to become colonized to the extent that said inoculated lignocellulose based medium is transformed into a fungal mycelium without any secondary organisms displacing the process through infection;

F—strictly regulating environmental conditions surrounding the lignocellulose based medium during said inoculation step and allowing step;

G—adding a primary compressive pressure on the lignocellulose based medium of at least 100 PSI;

H—reducing said primary compressive pressure; and

I—drying said colonized fungal mycelium for a specific time period.

Also this patent—even though it has numerous features that leave the impression of being very specific—has a obviously broad scope, since the specific parameters (e.g. the way the inoculated lignocellulose based medium is formed and the pressure of at least 100 PSI) appear to be so common that the subject matter of claim 1 of US307 may be argued to be not novel, for example over US5074959A, or at least not inventive. Note, part B is so vague that all and any white, brown and soft rot species could arguably fall under the patent.

### EP2702137B1 (EP137, see Table 4)

Granted claim 1 specifies the following:

A—A method of making dehydrated mycelium elements comprising the steps of:

B—creating a living hydrated mycelium composite containing at least one of a combination of mycelium and fibers, mycelium and particles, and mycelium, particles and fibers;

C—adding a nutrient material to said mycelium composite in an amount to promote mycelia tissue growth;

D—thereafter dehydrating the mycelium composite to a moisture content of less than 50 % by weight to deactivate the further growth of mycelia tissue to form a dehydrated mycelium element;

E—thereafter storing the dehydrated mycelium element at a temperature in the range of from − 50 °F to +200 ºF (− 45.56 to 93.33 ºC); and

F—adding moisture to said mycelium element in an amount sufficient to rehydrate said mycelium element and to re-activate mycelium on the exterior of said mycelium for growth into an adjacent mycelium element to bond said elements together to form a fabricated section; –OR.

G—creating a living hydrated mycelium composite containing at least one of a combination of mycelium and fibers, mycelium and particles, and mycelium, particles and fibers;

H—adding a nutrient material to said mycelium composite in an amount to promote mycelia tissue growth;

I—thereafter dehydrating the mycelium composite to a moisture content of less than 50% by weight to deactivate the further growth of mycelia tissue;

J—processing the dehydrated mycelium composite into a plurality of discrete particles; and

K—adding moisture to said plurality of discrete particles in an amount sufficient to re-hydrate said discrete particles and to re-activate mycelium on the exterior of said discrete particles for growth into adjacent discrete particles.

We argue that while this patent could conceivably be viewed as technical and specific to a general reader, from a mycological perspective it is clearly so vague and expansive to warrant concern. On the one hand, it includes two sets of features joined by an “OR”, so, it is in effect two alternative claims in one. On the other hand, the specific parameters, like the moisture content being reduced to less than 50% appear to be so common that the subject matter of claim 1 of US137 may be argued to be not novel, for example over ‘*Die Champignonkultur, Grundlagen und Fortschritte im Garten- und Weinbau* ‘ from 1958 [19], or at least not inventive. Other aspects raise questions- the intention to ‘create a living hydrated mycelium composite’ implies that a mycelium (or composite therefore) can be ‘created’ from a dead, dehydrated fungus. Given that mycelium-composites must by definition be generated from living and hydrated fungi, the adjectives ‘living hydrated’ are essentially redundant. Why are they included in the patent?

Taken together, our analysis of the patent situation in the field of material-oriented applications of fungal mycelium suggests that some patents do not clearly refer to new inventions with significant technological advancements. Some claims are trivial and refer to tacit knowledge in the field of mushroom farming and mycelium technology (e.g. from the former German Democratic Republic). But how to deal with patent (over-)protection?

In general, patent rights can always be fought in court, and there are several legal regulations for contesting and defending patent rights. In addition, other actions can be taken to overcome hindrance of innovation. We have summarized in Table 10 possible actions that can be taken and discuss some of their pros and cons.

**Table 10:** Options for (i) contesting patents and patent applications and (ii) and joint agreements

Action	Description	Pro	Con
Filing third party observations	Providing relevant information (e. g. prior art references) to the patent office before grant	• Very low cost and low effort if relevant prior art is already known • Anonymous filing may be possible • Offered at least with EPO, USPTO, UKIPO, and CIPO; • Outcome known before granting (on non-granting) of the patent	• Narrow time frame (between publication of the application and grant) • Outcome unclear • May not be possible in some jurisdictions
Filing an opposition	Challenging the patent in post-grand proceedings before the patent office	• Costs comparatively low compared to a nullity suit • Patent offices that have an opposition period include EPO (9 months), JPO (6 months), IP Australia (3 months), and others; • For bundle patents (like EP, even with opt-out regarding UPC) this affects the whole bundle	• Needs to be filed during the defined post-grand opposition period • Outcome unclear • Costs comparatively high (compared to third party observations) • Usually takes years until final decision
Filing a nullity suit	Challenging the patent before a local court or before the Unified Patent Court (UPC)	• Should always be possible, in all jurisdictions and (with possible exceptions) during the whole lifetime of the patent	• High costs • Outcome unclear • Usually takes years until final decision
Cooperation	Negotiating a cooperation between two or more parties	• Terms of the cooperation may be geared towards the common interests of the parties	• May require extensive negociations • In an unbalanced situation, it may be challenging to come to an agreement
Cross-licensing	Two (or more) parties agree to exchange licenses for the use of each other's patented technologies	• Low-cost (no fees for courts, etc.)	• Requires that both parties hold patents • May be considered unsatisfactory when only one of the exchanged patents is thought to be invalid
Licensing	Obtaining a license from the patent owner for the use of the patented technology	• Well-defined costs	• Unsatisfactory when the patent is thought to be invalid
No-attack-agreement	Obtaining permission to use a patented technology for free, in return for the assurance that no invalidating action against the patent is taken	• Use is for free	• Requires convincing arguments that the patent would be invalidated if challenged • Usually silently negotiated between two parties

As discussed in Table 10, the first two comparatively low-level options are not accessible any more for EP2094856B1, US11277979B2, EP2702137B1, and US9951307B2. Considering the preliminary opinion of the EPO Board of Opposition, chances of winning a nullity suit may be reasonable, but costs for filing and pursuing such a suit would have to be provided up-front, which may exceed financial resources of at least some of those who could potentially benefit from a less severe over-protection in the field. However, such a course of action could be possible in a collaborative community effort.

## Conclusions

The patent landscape of fungal-based materials shows that only a few key players have dominated the field right from the beginning. We argue that the current patent situation may have hindered and hinders other institutions and companies to innovate the field. At least for some of the patents causing over-protection, we consider the validity of the patents questionable as they refer to common and tacit knowledge in the mycology community. This may open up possibilities for nullity suits and/or negotiations for cooperation.

## Methods

*Patent search.* The databases Espacenet of the European Patent Office and DEPATISnet of the German Patent and Trademark Office were used for the patent search. The following search term was used in Espacenet: (nftxt = “mycelium” OR nftxt =  “mycelial”) AND (nftxt = “substrate” OR nftxt = “carrier”) AND (nftxt = (“composite” prox/distance < 3 “material”) OR nftxt = (“mycelial” prox/distance < 3 “composite”) OR nftxt = (“mycelium” prox/distance < 3 “scaffolding”) OR nftxt = (“building” prox/distance < 3 “material”) OR nftxt = (“biodegradable” prox/distance < 3 “material”)) AND (pn any “B1” OR pn any “B2”), and a corresponding term using the appropriate syntax was used for DEPATISnet.

The search result was re-examined by hand to exclude patents related to food, pharmaceutical applications (mainly production of antibiotics), fungicides, and other patents that, despite having the keywords, do not fall under the scope of mycelium-based materials for technical applications. The remaining patents were further examined regarding their subjects and actual claims. For retrieving the patent expiration date, Google Patents was used. If a patent family was incompletely reproduced by the search results, the patent family was completed from any of the databases. The patents resulting from the search were grouped topic- and family-wise. In Tables 4, 5, 6, 7 and 8, registered information included patent number, publication date, assignee/owner, title, status (including expiration date and countries where the patent is still pending), and claim 1.

### Supplementary Information

Additional file 1. Formal and substantive overview for patents given in Tables 4, 5, 6, 7 and 8.

Additional file 2. Features of the family member patents of EP2094856B1 (EP856).

## Data Availability

All data generated or analysed during this study are included in this published manuscript and its supplementary information file.
