# Supplementary material for: Patent landscape analysis for materials based on fungal mycelium: a guidance report on how to interpret the current patent situation
Source: Fungal Biol Biotechnol. 2024 Aug 10;11:11. doi: 10.1186/s40694-024-00177-2 (PMC11316976; doi:10.1186/s40694-024-00177-2)
Supplement: Supplementary file 1 — Additional file 1. Formal and substantive overview for patents given in Tables 4, 5, 6, 7 and 8. [file 40694_2024_177_MOESM1_ESM.docx]

**Additional File 1:** **Formal and substantive overview for patents given in Tables 4 to 8.** The Formal Overview provides formal information for the family members such as publication number and date, applicant, title, expiry date of the IP right and a link to the original document (only for the index patent). The Substantive Overview provides a brief overview of the subject matter of the paten including a relevant claim (usually claim 1) and a comment. Even though the patents and (family member) patent applications have been assigned to one of Tables 4 to 8 according to their area of use, it should be clear that the patent protected or claimed subject matters may not be so clearly separable. For example, a mycelium-based insulating material may be used both for insulating a house (Building/Construction Materials) and for insulating a refrigerator (Other Fungal Materials). An index application (shown with a white background) is listed for each patent family (typically the priority application or, if this is a US provisional application, the first regular application or the first granted application). If there are other family members, these are also listed after the main application with a light grey background. Patents having an unfavorable broad scope of protection are marked in the Patent Number field with **!!!**.

**Building/Construction Materials (corresponding to Table 4)**

Formal Overview:

| Patent/Application Number | Publica-tion Date | Applicant/ Owner | Title | Status Patent: Country and Expiry Date of Patent;  Application: Country in which grant is still possible; or E(xpired); | Link to original document |
| --- | --- | --- | --- | --- | --- |
| US11293005B2 | 05.04.2022 | ECOVATIVE DESIGN LLC, US | Process for making mineralized mycelium scaffolding and product made thereby | US  19.08.2039 | [US'300](https://depatisnet.dpma.de/DepatisNet/depatisnet?action=pdf&docid=US000011293005B2&xxxfull=1) |
| PCT/US2019/217175 | 14.11.2019 | see above | see above | E  (no national phase (NP) or regional phase (RP)) |  |
| US10604734B2 | 31.03.2020 | UNIV OF ALASKA ANCHO-RAGE, US | Thermal insulation material from mycelium and forestry byproducts | US 27.01.2037 | [US'734](https://depatisnet.dpma.de/DepatisNet/depatisnet?action=pdf&docid=US000010604734B2&xxxfull=1) |
| PCT/US2017/015359 | 03.08.2017 | see above | see above | E  (no NP/RP) |  |
| CN108699507A | 23.10.2018 | see above | see above | CN |  |
| US20200255794A1 | 13.08.2020 | see above | see above | US |  |
| KR101933573B1 | 28.12.2018 | KANG EUN CHANG, KR; SEO JUN WON, KR | Method for manufacturing a human-friendly functional panels using biopolymers | KR  22.10.2038 | [KR'573](https://depatisnet.dpma.de/DepatisNet/depatisnet?window=1&space=menu&content=treffer&action=pdf&docid=KR000101933573B1&xxxfull=1) |
| US9410116B2 | 09.08.2016 | MYCOWORKS INC, US; ROSS PHILIP, US | Method for producing fungus structures | US  23.09.2032 | [US'116](https://depatisnet.dpma.de/DepatisNet/depatisnet?action=pdf&docid=US000009410116B2&xxxfull=1) |
| US9951307B2 | 24.04.2018 | see above | see above | US  18.12.2031 |  |
| PCT/US2011/062264 | 31.05.2012 | see above | see above | E  (NP only for MX, abandoned) |  |
| CN103547668A | 29.01.2014 | see above | see above | CN |  |
| US10947496B2 | 16.03.2021 | see above | see above | US  28.11.2031 |  |
| US20210198621A1 | 01.07.2021 | see above | see above | US |  |
| JP5922225B2 | 24.05.2016 | ECOVATIVE DESIGN LLC, US | Method for producing a member comprising a dehydrated mycelium, and a product produced thereby | JP  25.04.2032 | [JP'225](https://depatisnet.dpma.de/DepatisNet/depatisnet?action=pdf&docid=JP000005922225B2&xxxfull=1) |
| PCT/US2012/034960 | 01.11.2012 | see above | see above | NP CA, JP, AU;  RP EP |  |
| DK2702137T3 | 20.03.2017 | see above | see above | DK  25.04.2032 |  |
| **!!!**  EP2702137B1 | 05.03.2014 | see above | see above | DE, NL, DK, FR, GB  25.04.2032 |  |
| AU2012249802B2 | 04.08.2016 | see above | see above | AU  25.04.2032 |  |
| CA2834095C | 01.11.2012 | see above | see above | CA  25.04.2032 |  |

Substantive Overview:

| Patent/Application Number | Claim | Comment |
| --- | --- | --- |
| US11 293005B2 | 1. A structure comprising  a scaffold of fungal biopolymer of predetermined form characterized in being formed of a network of interconnected mycelia cells; and  **a coating of at least one of an apatite, a carbonate, and a silicate** on at least some of said cells within said network. | Production of a mycelium with subsequent mineralisation |
| US10 604734B2 | 1. A biodegradable insulation material comprising  a. a structural scaffold comprising a three-dimensional structure and a **mycelium from a first temperature resilient fungus**, wherein the mycelium from the first temperature resilient fungus has colonized the three-dimensional structure, wherein the structural scaffold has a chitinous hydrophobic outer skin; and  b. a substrate comprising nutritive media and a **mycelium from a second temperature resilient fungus**, wherein the mycelium from the second temperature resilient fungus has colonized the substrate;  wherein the biodegradable insulation material is the result of the structural scaffold and substrate fusing together,  wherein **the first temperature resilient fungus and second temperature resilient fungus are different**. | Insulating material that uses two different mycelia and is advantageous for use in cold, structurally weak regions (e.g. Alaska - see Applicant), for example, when insulating houses, etc. |
| CN108699507A | 1. A kind of biodegradable isolated material comprising  A. structure stand, the structure stand include the nutrient medium for fungal mycelium; with  B. at least one thermal adaptability fungi. | The current claim is significantly **broader** than the claim granted for the main application.  A restriction in the granting procedure does not seem unlikely. |
| US20200255794A1 | 1. A biodegradable insulation material comprising  a. a structural scaffold comprising a three-dimensional structure and a mycelium from a first temperature resilient fungus, wherein the mycelium form the first temperature resilient fungus has colonized the three-dimensional structure, wherein the structural scaffold has a chitinous hydrophobic outer skin; and  b. a substrate comprising nutritive media and a mycelium from a second temperature resilient fungus, wherein the mycelium from the second temperature resilient fungus has colonized the substrate;  wherein the biodegradable insulation material is the result of the structural scaffold and substrate fusing together,  **wherein the first temperature resilient fungus and second temperature resilient fungus are the same.** | In contrast to US10 604734B2, both temperature-resistant fungi are **the same** here. |
| KR101 933573B1 | A method for producing a functional plate material using biomass resources, which are either discarded or unused, and **biopolymer mushroom mycelium**  Collecting the biomass and sterilizing it to obtain sterilized biomass; and  Culturing a biopolymer spore based on porphyrobacteria on a sterilized PDA medium;  Preparing a liquid type biopolymer based or solid type biopolymer based on a part of the cultured biopolymer spores;  A first mixture obtaining step of adding and mixing the sterilized biomass and the biopolymer base material with nutrients and moisture to obtain a first mixture;  A first growing step of adding the first mixture into a mold and growing the mixture;  And a secondary growth step of removing the forming mold and growing the first primary growth material as a secondary growth material. The method of claim 1, wherein  And a step of subjecting the secondarily grown plate shaped material to a pressure treatment at a temperature of 110 to 150 ° C at 0 to 10 kgf / cm 2 for 10 to 60 minutes using a hot press, thereby producing a low density functional sheet as a heat insulating material or a sound absorbing material Wherein the biopolymer is a biodegradable polymer. | It is unclear which type of mushroom is used here, as the mushroom is only referred to as "biopolymer mushroom".  It is also not entirely clear whether a fungus is used at all, as bacteria are also mentioned.  However, this non-patent publication, for example: https://www.mdpi.com/1996-1944/14/11/2906  the shiny lacquered mushroom (Ganoderma lucidum) is used as a "biopolymer mushroom" in combination with bacteria. |
| US9 410116B2 | A method for growing organically derived building material in the form of a moldable substrate to serve a wide range of manufacturing and construction applications, the method comprising the steps of:  a) obtaining a lignocellulose based medium capable of supporting the growth of saprophytic fungi;  b) mixing said lignocellulose based medium with water to reach a hydration level;  c) inoculating said lignocellulose based medium with a fungal inoculum;  d) allowing time for said inoculated lignocellulose based medium to become colonized to the extent that said inoculated lignocellulose based medium is transformed into a fungal mycelium without any secondary organisms displacing the process through infection;  e) providing a vessel in which said allowing step occurs and wherein environmental conditions in said vessel are regulated;  f) **placing said fungal mycelium into a mold such that the fungal mycelium forms into a fungal molded shape;**  g**) applying a primary compressive pressure of at least 100 PSI to the lignocellulose based medium, wherein before applying the primary compressive pressure, a plurality of rods and a plurality of slats of organic material-are layered near the top and the bottom of surface substrate, where each of the plurality of rods and the plurality of slats of organic material—and are positioned at right angle to each other to increase the structural capacities of the organically derived material;**  **h) reducing said primary compressive pressure by a factor of at least 4;**  **i) removing said rod and slat embedded fungal molded shape from said mold**; and  j) drying said fungal molded shape at a specific temperature for a specific time period. | The process involves allowing a substrate to grow through a substrate, followed by compression and drying.  A characteristic feature appears to be process steps f) to i), in which **rods and plates** are inserted into the mycelium before pressing and removed again after pressing |
| **!!!**  US9 951307B2 | A method for growing organically derived building materials in the form of a moldable substrate to serve a wide range of manufacturing and construction applications, the method comprising the steps of:  a) obtaining a lignocellulose based medium capable of supporting the growth of saprophytic fungi;  b) mixing said lignocellulose based medium with water to reach a hydration level;  c) inoculating said lignocellulose based medium with a fungal inoculum;  d) allowing time for said inoculated lignocellulose based medium to become colonized to the extent that said inoculated lignocellulose based medium is transformed into a fungal mycelium without any secondary organisms displacing the process through infection;  e) strictly regulating environmental conditions surrounding the lignocellulose based medium during said inoculation step and allowing step;  **f) adding a primary compressive pressure on the lignocellulose based medium of at least 100 PSI;**  g) reducing said primary compressive pressure; and  h) drying said colonized fungal mycelium for a specific time period. | Apart from the specified pressure, on the lignocellulose based medium, the patent claim does not appear to contain any specific process steps, despite step e) claiming to "strictly" regulate environmental conditions: without specific instructions as to which environmental conditions are to be controlled and in what range, this is too vague. |
| CN103547668A | 1.A method of growing an organically derived building material in the form of a moldable matrix that is widely used in manufacturing and construction applications, the method comprising the steps of:  a) obtaining a lignocellulosic medium capable of supporting the growth of saprophytic fungi;  b) mixing the lignocellulose-based medium with water to achieve a degree of hydration;  c) inoculating the lignocellulose-based medium with a fungal inoculum;  d) allowing time for the inoculated lignocellulose-based medium to colonize to the extent that the inoculated lignocellulosic medium is converted to fungal mycelium without any second organism replacing it by infection process;  e) providing a container in which the leaving step occurs and in which the environmental conditions in the container are adjusted;  f) placing the fungal mycelium into a mold such that the fungal mycelium forms a fungal molded body;  **g) applying a first compression pressure of at least 100 PSI to the lignocellulosic medium;**  **h) reducing the first compression pressure to at least 1/4;**  i) removing the fungal molded body from the mold after the placing step;  j) drying the fungus at a given temperature to mold the body for a given period of time. | Specific pressure and reducing of the pressure to a defined fraction of the original pressure. |
| US10947496B2 | 1. A molding system for forming an inoculated lignocellulose based medium into a fungal molded shape, the molding system comprising:  a. an inoculated lignocellulose based medium;  b. a vessel within which environmental conditions are regulated, the vessel comprising the inoculated lignocellulose based medium that is saturated with water, the inoculated lignocellulose based medium further comprising nitrogen, trace elements, and a buffer to balance the pH of the inoculated lignocellulose based medium;  c. the vessel capable of supporting growth of saprophytic fungi without any secondary organisms displacing the process through infection;  d. **a secondary organic material layered near the top and bottom of the inoculated lignocellulose based medium, the secondary organic material comprising a cross-grained laminate of at least two layers comprising components set at right angles relative to one another**;  e. **a hard mold containing the flexible vessel; and**  **f. a compressive system for applying a primary compressive pressure of at least 10 PSI to the lignocellulose based medium and a secondary compressive pressure of between 300-500 psi to the lignocellulose based medium such that at least some water is forced out of the medium, allowing it to absorb an agent and take on a fungal molded shape; wherein the compressive system further comprise an aqueous gel agent to be absorbed within the fungal mycelium to form the fungal molded shape; and**  **g. a filter for filtering the inoculated lignocellulose based medium.** | Characteristic features appear to be process steps d) to h), with the design of the pressure vessel, the pressure curve, etc. |
| US20210198621A1 | 1. A molding system comprising:  a. a first inoculated lignocellulose based medium;  b. the first inoculated lignocellulose based medium comprising nitrogen, trace elements, and **a buffer to balance the pH** of the first inoculated lignocellulose based medium;  c. a second, grown inoculated lignocellulose based medium;  d. a secondary material layered between said first and second media, the secondary material comprising a cross-grained laminate of at least two layers comprising components set at right angles relative to one another;  e. a filter for filtering the inoculated lignocellulose based medium; and  f. a compressive system for applying a primary compressive pressure of at least 10 PSI to the lignocellulose based medium and a secondary compressive pressure **of between 300-500 psi** to the lignocellulose based medium such that at least some water is forced out of the medium, allowing it to absorb an agent and take on a fungal molded shape;  **g. whereby said second, grown inoculated lignocellulose based medium contains in it a cellulosic or non-cellulosic fabric.** | In contrast to the other members of the family, **fabric** is introduced into the mycelium here. |
| JP59 22225B2 | 1. Creating a live hydrated mycelium composite material comprising at least one of mycelium and fibers, mycelium and particles, and combinations of mycelium, particles and fibers; Adding the nutrient material to the composite material in an amount to promote growth of mycelium tissue, and then **dehydrating the live hydrated mycelium composite material to a water content of less than 50% by weight** to Deactivating further growth of the body tissue to form a dehydrated mycelium member, **adding water to the dehydrated mycelium member to reactivate and grow the mycelium outside the dehydrated mycelium member Forming an assembly by integrally joining a mycelium member to an adjacent mycelium member via the grown mycelium**. | Specifically described drying and moistening processes with coalescence of neighbouring mycelia |
| **!!!**  EP2702137B1 | A method of making dehydrated mycelium elements comprising the steps of:  (1) creating a living hydrated mycelium composite containing at least one of a combination of mycelium and fibers, mycelium and particles, and mycelium, particles and fibers;  adding a nutrient material to said mycelium composite in an amount to promote mycelia tissue growth;  thereafter dehydrating the mycelium composite to a moisture content of less than 50 % by weight to deactivate the further growth of mycelia tissue to form a dehydrated mycelium element;  thereafter storing the dehydrated mycelium element at a temperature in the range of from -50 °F to +200 ºF (-45.56 to 93.33 ºC); and  adding moisture to said mycelium element in an amount sufficient to rehydrate said mycelium element and to re-activate mycelium on the exterior of said mycelium for growth into an adjacent mycelium element to bond said elements together to form a fabricated section;  **OR**  (2) creating a living hydrated mycelium composite containing at least one of a combination of mycelium and fibers, mycelium and particles, and mycelium, particles and fibers;  adding a nutrient material to said mycelium composite in an amount to promote mycelia tissue growth;  thereafter dehydrating the mycelium composite to a moisture content of less than 50% by weight to deactivate the further growth of mycelia tissue;  processing the dehydrated mycelium composite into a plurality of discrete particles; and  **adding moisture to said plurality of discrete particles in an amount sufficient to re-hydrate said discrete particles and to re-activate mycelium on the exterior of said discrete particles for growth into adjacent discrete particles.** | Even though claim 1 looks long and fairly detailed/specific, the method steps appear to be very basic and the specified parameter ranges very broad, and claim 1 includes two alternatives.  As a consequence, this claim may possibly be relevant for fairly basic processing.  Each of the the methods may have been employed in **edible mushroom production**, which has a comparatively long tradition. See discussion section of the paper. |
| AU2012249802B2 | A method of making dehydrated mycelium elements comprising the steps of creating a living hydrated mycelium composite containing at least one of a combination of mycelium and fibers, mycelium and particles, and mycelium, particles and fibers;  adding a nutrient material to said mycelium composite in an amount to promote mycelia tissue growth;  thereafter dehydrating the mycelium composite to a moisture content of less than 50% by weight to deactivate the further growth of mycelia tissue to form a dehydrated mycelium element;  thereafter storing the dehydrated mycelium element at a temperature in the range of from -50oF to +200oF; and adding moisture to said mycelium element in an amount sufficient to re hydrate said mycelium element and to re-**activate mycelium on the exterior of said mycelium for growth into an adjacent mycelium element to bond said elements together to form a fabricated section.** | Drying and moistening processes with further growth and coalescence of neighbouring mycelia |
| CA2834095C | 1. A method of making a dehydrated mycelia composite comprising the steps of forming an inoculum including a preselected fungus; forming a mixture of a substrate of discrete elements and a nutrient material, said nutrient material being capable of being digested by said fungus; adding said inoculum to said mixture; allowing said fungus to digest said nutrient material in said mixture over a period sufficient to grow hyphae and to allow said hyphae to form a network of interconnected mycelia cells through and around said discrete elements thereby bonding said discrete elements together to create a living hydrated mycelia composite; and thereafter dehydrating the mycelia composite to a moisture content of less than 30% by weight at a temperature less than 140 F to deactivate the further growth of mycelia tissue and to maintain the mycelia therein in a de-activated state **capable of being re-activated for growth upon the addition of moisture.** | Drying and moistening processes with further growth and coalescence of neighbouring mycelia |

**Textile Materials (corresponding to Table 5)**

Formal Overview:

| Patent/Application Number | Publication Date | Applicant/ Owner | Title | Status Patent: Country and Expiry Date of Patent;  Application: Country in which grant is still possible; or E(xpired); | Link to Original document |
| --- | --- | --- | --- | --- | --- |
| PCT/US2020/038194 | 24.12.2020 | THE FYNDER GROUP INC, US | Fungal textile materials and leather analogs | NP:CN, US, KR, JP, CA, TW  RP: EP | [PCT'194](https://depatisnet.dpma.de/DepatisNet/depatisnet?action=pdf&docid=WO002020257320A1&xxxfull=1) |
| US11118305B2 | 14.09.2021 | see above | see above | US  17.06.2040 |  |
| US11414815B2 | 16.08.2022 | see above | see above | US  17.06.2040 |  |
| US11447913B2 | 20.09.2022 | see above | see above | US  17.06.2040 |  |
| US11427957B2 | 30.08.2022 | see above | see above | US  17.06.2040 |  |
| US11643772B2 | 09.05.2023 | see above | see above | US  17.06.2040 |  |
| US11649586B2 | 16.05.2023 | see above | see above | US  21.07.2041 |  |
| US11718954B2 | 08.08.2023 | see above | see above | US  17.06.2040 |  |
| US11952713B2 | 09.04.2024 | see above | see above | US  17.06.2040 |  |

Substantive Overview:

| Patent/Application Number | Claim | Comment |
| --- | --- | --- |
| PCT/US2020/038194 | A method for preparing a durable **sheet material** comprising fungal biomass, comprising:  (a) causing a solution to infiltrate an inactivated fungal biomass, the solution comprising a **solvent and a component selected from the group consisting of a polymer, a crosslinker**, and combinations and mixtures thereof; and  (b) curing the biomass to remove solvent from the biomass and form the durable sheet material. | Production of a hard-wearing layered material as a combination of fungal biomass and a polymer. |
| US11118305B2 | A method for making a durable sheet material, comprising:  (a) contacting an inactivated fungal biomass with an aqueous solution comprising a liming substance;  (b) contacting the inactivated fungal biomass from step (a) with an aqueous solution comprising a deliming substance;  (c) contacting the inactivated fungal biomass from step (b) with an aqueous solution comprising a **polymer**;  (d) contacting the inactivated fungal biomass from step (c) with an **aqueous solution comprising a crosslinker**;  (e) contacting the inactivated fungal biomass from step (d) with an aqueous solution comprising a **plasticizer**;  (f) drying the inactivated fungal biomass from step (e) to form a dried inactivated fungal biomass; and  (g) heat-pressing the dried inactivated fungal biomass to form the durable sheet material. | Production of a hard-wearing layered material as a combination of fungal biomass and a polymer. |
| US11414815B2 | 1. A method for preparing a durable sheet material comprising fungal biomass, comprising:  (a) causing an aqueous polymer solution, comprising a **solvent and a polymer**, to infiltrate an inactivated fungal biomass to a fungal biomass:**polymer loading ratio of between 25:75 and 75:25**; and  (b) curing the biomass to remove solvent from the biomass and form the durable sheet material,  wherein the inactivated fungal biomass comprises a cohesive fungal biomass. | Ratio of fungal biomass to polymer is defined |
| US11447913B2 | A textile composition, comprising:  an inactivated fungal biomass; and  a polymer,  wherein the polymer is infiltrated into the inactivated fungal biomass to a fungal biomass:polymer loading ratio of between about 25:75 and about 75:25, and  wherein the textile composition has a tensile strength a at least about 2 MPa. | Ratio of fungal biomass to polymer is defined |
| US11427957B2 | A method for making a durable sheet material, comprising:  (a) inactivating a fungal biomass by boiling the biomass in water;  (b) contacting the inactivated fungal biomass with an aqueous solution comprising calcium hydroxide to form a limed inactivated fungal biomass;  (c) contacting the limed inactivated fungal biomass with an aqueous solution comprising **ammonium sulfate** to form a delimed inactivated fungal biomass;  (d) contacting the delimed inactivated fungal biomass with an aqueous solution comprising an alkali metal halide to form a pickled inactivated fungal biomass;  (e) contacting the pickled inactivated fungal biomass with a **first crosslinker** to form a tanned inactivated fungal biomass;  (f) contacting the tanned inactivated fungal biomass with an aqueous solution comprising at least one of a second crosslinker and a polymer to form a re-tanned inactivated fungal biomass;  (g) contacting the re-tanned inactivated fungal biomass with a **fatliquoring oil to form a fatliquored inactivated fungal biomass;**  (h) adhering a non-fungal textile backing to the inactivated fungal biomass to form a backed inactivated fungal biomass;  (i) heat-pressing the backed inactivated fungal biomass to form a heat-pressed inactivated fungal biomass;  (j) drying the heat-pressed inactivated fungal biomass to form a dried inactivated fungal biomass; and  (k) applying at least one of a **finishing wax, a finishing oil, and nitrocellulos**e to the dried inactivated fungal biomass to form the durable sheet material. | Very detailed description of the method |
| US11643772B2 | A composite fungal sheet, comprising a **first inactivated fungal biomass** and a **second inactivated fungal biomass**, wherein:  the first inactivated fungal biomass comprises a first **polymer** and an intact filamentous fungal biomat;  the second inactivated fungal biomass comprises a second polymer and size-reduced filamentous fungal particles; and  the first and second inactivated fungal biomasses are **laminated together**. | Composite of two inactivated fungal biomasses |
| US11649586B2 | A method for preparing a durable **sheet** material comprising **fungal biomass**, comprising:  (a) causing a solution to infiltrate an inactivated fungal biomass, the solution comprising a solvent and a component selected from the group consisting of a polymer, a crosslinker, and combinations and mixtures thereof;  (b) curing the biomass to remove solvent from the biomass and form the durable sheet material; and  (c) at least one of (i) adding a **thermal dopant** to the inactivated fungal biomass and (ii) adding a thermal dopant to the durable sheet material after step (b). | Fungal biomass sheet with thermal dopant |
| US11718954B2 | A method for preparing a **leather analog** material comprising fungal biomass, comprising:  (a) size-reducing an inactivated cohesive fungal biomass;  (b) causing an aqueous solution, comprising a solvent, a polymer, and a plasticizer, to infiltrate the size-reduced inactivated fungal biomass to a fungal biomass:**polymer** loading ratio of between 25:75 and 75:25 by blending the size-reduced inactivated fungal biomass with the solution to form a blended composition;  (c) curing the biomass to remove solvent from the biomass and form the leather analog material, wherein the leather analog material has a tensile strength of at least about 4.5 MPa and a thickness of between about 0.5 mm and about 30 mm; and  (d) adhering a **non-fungal textile backing** to the biomass. | Leather analog with included polymer and textile backing |
| US11952713B2 | A textile composition, comprising:  an inactivated fungal mycelial biomass;  a non-fungal **textile backing**;  a **polymer**; and  a plasticizer;  wherein:  the polymer comprises a biopolymer,  the polymer is infiltrated into the inactivated fungal biomass to a fungal **biomass:polymer loading ratio** of about 25:75 to about 75:25, and  the textile composition has a **tensile strength** of at least about 2 MPa. | Includes textile backing, biopolymer, specific tensile strength |

**Filtration Materials (corresponding to Table 6)**

Formal Overview:

| Patent/Application Number | Publication Date | Applicant/ Owner | Title | Status Patent: Country and Expiry Date of Patent;  Application: Country in which grant is still possible; or E(xpired); | Link to Original document |
| --- | --- | --- | --- | --- | --- |
| US10087094B2 | 02.10.2018 | PONTIFICIA UNIV JAVERIANA, CO | Consortium of fungi immobilized on a laminar lignocellulose carrier for the treatment of wastewater and method for producing same | US  09.12.2034 | [US'094](https://depatisnet.dpma.de/DepatisNet/depatisnet?action=pdf&docid=US000010087094B2&xxxfull=1) |
| US9714180B2 | 25.07.2017 | BAYER EBEN, US; ECOVATIVE DESIGN LLC, US; MCINTYRE GAVIN, US; SCULLY CHRISTOPHER, US | Composite material for absorbing and remediating contaminants and method of making same | US  12.01.2034 | [US'180](https://depatisnet.dpma.de/DepatisNet/depatisnet?action=pdf&docid=US000009714180B2&xxxfull=1) |

Substantive Overview:

| Patent/Application Number | Claim | Comment |
| --- | --- | --- |
| US10087094B2 | A laminar biocarrier for the treatment of wastewaters with an **elastic, flexible and resistant mesh shape**, wherein said laminar biocarrier is prepared by weaving or interlacing lignocellulosic yarns, and holds and immobilizes a **wood-decay fungi biomass layer**. | A fungal biomass layer is fixed onto a grid-shaped bio-support for wastewater treatment |
| US9714180B2 | 1. A composite material comprising a **mass of pellets, each said pellet composed of a saprophytic fungi strain characterized in producing an enzyme capable of breaking down animal waste and a particulate material wherein said fungi forms a plurality of hyphae bonded to said particulate material.** | Mushroom pellets that are able to process animal faeces. |

**Chitosan Material (corresponding to Table 7)**

Formal Overview:

| Patent/Application Number | Publication Date | Applicant/ Owner | Title | Status Patent: Country and Expiry Date of Patent;  Application: Country in which grant is still possible; or E(xpired); | Link to Original document |
| --- | --- | --- | --- | --- | --- |
| US9982393B2 | 29.05.2018 | WESTERN MICHIGAN UNIV RESEARCH FOUNDATION, US | Chitosan as a biobased barrier coating for functional paperboard products | US  28.11.2036 | [US'393](https://depatisnet.dpma.de/DepatisNet/depatisnet?action=pdf&docid=US000009982393B2&xxxfull=1) |

Substantive Overview:

| Patent/Application Number | Claim | Comment |
| --- | --- | --- |
| US9982393B2 | A composite fiber stock material comprising:  at least one layer of a fibrous base sheet; and  at least one **chitosan layer** comprising a chitosan coat weight from about 1 g/m2 to about 10 g/m2;  wherein the composite fiber stock material has an air permeance from about 20 nm/Pa s to about 50 nm/Pa s.aerating the aqueous solution after a suitable exposure time to the chlorine dioxide, wherein the aerating halts the adverse effect of the chlorine dioxide on the microorganism biomass autolysate; and  providing a volume of an organic solvent to float on top of the aqueous solution, after said treating, wherein the volume of the organic solvent is sufficient to dissolve the lipids of the autolysate;  wherein said providing the volume of the organic solvent causes the autolysate to separate into an upper layer comprising the organic solvent and the lipids; and a lower layer comprising the aqueous solution and non-lipid materials of the autolysate;  wherein said upper layer protects said lower layer from environmental microbial contamination. | Chitosan production from mycelium. |

**Other Materials (corresponding to Table 8)**

Formal Overview:

| Patent/Application Number | Publication Date | Applicant/ Owner | Title | Status Patent: Country and Expiry Date of Patent;  Application: Country in which grant is still possible; or E(xpired); | Link to Original document |
| --- | --- | --- | --- | --- | --- |
| US11638671B2 | 02.05.2023 | TRAMMELL JENNIFER, US | Mycelium composite burial container | US  10.09.2041 | [US'671](https://depatisnet.dpma.de/DepatisNet/depatisnet?action=pdf&docid=US000011638671B2&xxxfull=1) |
| US11359074B2 | 14.06.2022 | ECOVATIVE DESIGN LLC, US | Solution based post-processing methods for mycological biopolymer material and mycological product made thereby | US  12.08.2038 | [US'074](https://depatisnet.dpma.de/DepatisNet/depatisnet?action=pdf&docid=US000011359074B2&xxxfull=1) |
| JP7161489B2 | 26.10.2022 | see above | see above | JP  29.03.2038 |  |
| AU2018243372A1 | 31.10.2019 | see above | see above | AU |  |
| CA3058212A1 | 04.10.2018 | see above | see above | CA |  |
| EP3599832A4 | 27.01.2021 | see above | see above | All EP states |  |
| CN110506104A | 26.11.2019 | see above | see above | CN |  |
| PCT/US2018/025235 | 04.10.2018 | see above | see above | NP CA, CN, JP, BR, AU, NZ, EP, IN |  |
| US20230013465A1 | 19.01.2023 | see above | see above | US |  |
| BR112019020132A8 | 14.03.2023 | see above | see above | BR |  |
| US11310968B2 | 26.04.2022 | MYCOWORKS INC, US | System for growing fungal materials | US  14.07.2037 | [US'968](https://depatisnet.dpma.de/DepatisNet/depatisnet?action=pdf&docid=US000011310968B2&xxxfull=1) |
| EP3484995A4 | 22.01.2020 | see above | see above | All EP states |  |
| MX2019386A | 18.09.2019 | see above | see above | MX |  |
| PCT/US2017/042267 | 18.01.2018 | see above | see above | MX, EP |  |
| US10842089B2 | 24.11.2020 | see above | see above | US  21.11.2038 |  |
| US11013189B2 | 25.05.2012 | see above | see above | US  14.07.2037 |  |
| US11310968B2 | 26.04.2022 | see above | see above | US  14.07.2037 |  |
| US20220279736A1 | 08.09.2022 | see above | see above | US |  |
| US11359174B2 | 14.06.2022 | ECOVATIVE DESIGN LLC, US | Bioreactor paradigm for the production of secondary extra-particle hyphal matrices | US  01.05.2040 | [US'174](https://depatisnet.dpma.de/DepatisNet/depatisnet?action=pdf&docid=US000011359174B2&xxxfull=1) |
| PCT/US2019/047977 | 09.04.2020 | see above | see above | NP: AU, NZ, CA, EP |  |
| AU2019352842A1 | 15.04.2021 | see above | see above | AU |  |
| CA3113935A1 | 09.04.202 | see above | see above | CA |  |
| EP3860370A4 | 12.10.2022 | see above | see above | EP |  |
| US20230056666A1 | 23.02.2023 | see above | see above | US |  |
| **!!!**  US11277979B2 | 22.03.2022 | ECOVATIVE DESIGN LLC, US | Mycological biopolymers grown in void space tooling | US  15.06.2036 | [US'979](https://depatisnet.dpma.de/DepatisNet/depatisnet?action=pdf&docid=US000011277979B2&xxxfull=1) |
| US20220290199A1 | 15.09.2022 | see above | see above | US |  |
| US10945382B2 | 16.03.2021 | GROWSQUARES INC, US | Soil module and method of manufacture thereof | US  07.11.2038 | [US'382](https://depatisnet.dpma.de/DepatisNet/depatisnet?action=pdf&docid=US000010945382B2&xxxfull=1) |
| US11102938B2 | 31.08.2021 | see above | see above | US  07.11.2038 |  |
| US11015059B2 | 25.05.2021 | BOLT THREADS INC, US | Composite material, and methods for production thereof | US  22.05.2040 | [US'059](https://depatisnet.dpma.de/DepatisNet/depatisnet?window=1&space=menu&content=treffer&action=pdf&docid=US000011015059B2&xxxfull=1) |
| PCT/US2020/034354 | 26.11.2020 | see above | see above | NP: SG, CA, AE, MX, TH, CN, JP, BR, AU, EP, RU, KR |  |
| US20210292706A1 | 23.09.2021 | see above | see above | US |  |
| AU2020279832A1 | 06.01.2022 | see above | see above | AU |  |
| CA3137693A1 | 26.11.2020 | see above | see above | CA |  |
| CN114127278A | 01.03.2022 | see above | see above | CN |  |
| EP3973055A4 | 07.06.2023 | see above | see above | all EP states |  |
| JP2022534025A | 27.07.2022 | see above | see above | JP |  |
| KR20220027075A; | 07.03.2022 | see above | see above | KR |  |
| MX2021014233A | 06.01.2022 | see above | see above | MX |  |
| SG11202112275VA | 30.12.2021 | see above | see above | SG |  |
| TW202112943A | 01.04.2021 | see above | see above | TW |  |
| **!!!**  EP2094856B1 | 15.06.2016 | RENSSELAER POLYTECHNIC INSTITUTE, ECOVATIVE DESIGN LLC, US | Method for producing grown materials and products made thereby | ES, PL, SI, CH, HU, BE, TR, SK, SE, RO, PT, NL, LV, LT, IT, IE, GB, FR, FI, DK, DE, BG, AT,  13.12.2027 | [EP'856](https://worldwide.espacenet.com/patent/search/family/039512340/publication/EP2094856B1?q=EP2094856B1) |
| PCT/US2007/025475 | 19.06.2008 | see above | see above | NP: EP JP CA NZ CN AU |  |
| US9485917B2 | 08.11.2016 | see above | see above | US  31.07.2035 | [US'917](https://depatisnet.dpma.de/DepatisNet/depatisnet?action=pdf&docid=US000009485917B2&xxxfull=1) |
| IL199315A | 30.11.2015 | see above | see above | IL  FD+20Y: 11.06.2029 |  |
| US8999687B2 | 07.04.2015 | see above | see above | US  18.05.2028 |  |
| US10525662B2 |  | see above | see above | US  27.03.2028 |  |
| US9801345B2 | 31.10.2017 | see above | Self-supporting composite material made with mycelia cells and method of making same | US  31.03.2030 |  |
| US10589489B2 | 17.03.2020 | see above | Composite material | US  11.10.2028 |  |
| US9795088B2 | 24.10.2017 | see above | Fabricated panel | US  03.02.2029 |  |
| JP5740492B2 | 24.06.2015 | see above | Method for producing growth material and product produced thereby | JP  13.12.2027 |  |
| IL234585A | 29.06.2017 | see above | Panels comprising a self-supporting composite material and a veneer material | IL  FD+20Y: 11.09.2034 |  |
| IL234584B | 28.02.2018 | see above | Self-supporting composite material comprising a substrate of discrete particles and a network of interconnected mycelia cells | IL  FD+20Y: 11.09.2034 |  |
| US10589489B2 | 17.03.2020 | see above | Composite material | US  11.10.2028 |  |
| US10583626B2 | 23.02.2017 | see above | Method for producing grown materials | US  28.08.2028 |  |
| US11932584B2 | 19.03.2024 | see above | Method of Forming a Mycological Product | US  21.05.2028 |  |
| AU2007333545B2 | 15.08.2013 | see above | Method for producing grown materials and products made thereby | AU  13.12.2027 |  |
| CA2672312C | 06.01.2015 | see above | Method for producing grown materials and products made thereby | CA  13.12.2027 |  |
| CN101627127B | 27.05.2015 | see above | see above | CN  13.12.2027 |  |
| KR102001771B1 | 18.07.2019 | KOREA MARITIME UNIV IND ACAD, KR | Manufacturing method for eco-friendly working materials with coffee waste | KR  12.02.2038 | [KR'771](https://depatisnet.dpma.de/DepatisNet/depatisnet?action=pdf&docid=KR000102001771B1&xxxfull=1) |
| US8313939B2 | 20.11.2012 | FORD GLOBAL TECH INC, US; JOHNSTON TENGLER ELIZABETH CLEARY, US; KALISZ RAYMOND EDWARD, US; PETRELLA-LOVASIK ROSARIA LEE, US; ROCCO CHARLES ALAN, US | Injection molded mycelium and method | US  29.12.2030 | [US'939](https://depatisnet.dpma.de/DepatisNet/depatisnet?action=pdf&docid=US000008313939B2&xxxfull=1) |
| US8298809B2 | 30.10.2012 | FORD GLOBAL TECH LLC, US; KALISZ RAYMOND EDWARD, US; ROCCO CHARLES ALAN, US | Method of making a hardened elongate structure from mycelium | US  13.08.2030 | [US'809](https://depatisnet.dpma.de/DepatisNet/depatisnet?action=pdf&docid=US000008298809B2&xxxfull=1) |
| US8298810B2 | 30.10.2012 | FORD GLOBAL TECH LLC, US; KALISZ RAYMOND EDWARD, US; ROCCO CHARLES ALAN, US | Mycelium structure with self-attaching coverstock and method | US  25.12.2030 | [US'810](https://depatisnet.dpma.de/DepatisNet/depatisnet?action=pdf&docid=US000008298810B2&xxxfull=1) |
| US8283153B2 | 09.10.2012 | FORD GLOBAL TECH LLC, US; KALISZ RAYMOND EDWARD, US; ROCCO CHARLES ALAN, US | Mycelium structures containing nanocomposite materials and method | US  09.06.2030 | [US'153](https://depatisnet.dpma.de/DepatisNet/depatisnet?action=pdf&docid=US000008283153B2&xxxfull=1) |
| US8227224B2 | 24.07.2012 | FORD GLOBAL TECH LLC, US; KALISZ RAYMOND EDWARD, US; ROCCO CHARLES ALAN, US | Method of making molded part comprising mycelium coupled to mechanical device | US  04.09.2030 | [US'224](https://depatisnet.dpma.de/DepatisNet/depatisnet?action=pdf&docid=US000008227224B2&xxxfull=1) |
| US8227225B2 | 24.07.2012 | FORD GLOBAL TECH LLC, US; KALISZ RAYMOND EDWARD, US; ROCCO CHARLES ALAN, US | Plasticized mycelium composite and method | US  01.09.2030 | [US'225](https://depatisnet.dpma.de/DepatisNet/depatisnet?action=pdf&docid=US000008227225B2&xxxfull=1) |
| US8227233B2 | 24.07.2012 | FORD GLOBAL TECH LLC, US; KALISZ RAYMOND EDWARD, US; ROCCO CHARLES ALAN, US | Method of making foamed mycelium structure | US  01.09.2030 | [US'233](https://depatisnet.dpma.de/DepatisNet/depatisnet?action=pdf&docid=US000008227233B2&xxxfull=1) |

Substantive Overview:

| Patent/Application Number | Claim | Comment |
| --- | --- | --- |
| US11638671B2 | A **container for holding a deceased** or their remains comprising:  a) an outer surface made at least in part of mycelium composite, wherein the outer surface comprises a **plurality of equally spaced apart grooves on at least a majority of the outer surface;**  b) an opening for receiving the deceased or their remains;  c) a lid made at least in part of mycelium composite, wherein the lid is configured to completely seal the opening; and  d) **one or more pegs made at least in part of mycelium composite**, each peg comprising a first end and a second end distal from the first end, the first end configured for insertion into any of the plurality of grooves, the second end consisting essentially of a decorative element. | Coffin made of mushroom mycelium, in which not only the base and lid, but **also at least some of the pegs are made of mycelium material** |
| US11359074B2 | A method comprising the steps of:  obtaining a tissue consisting essentially of fungal mycelium containing native moisture, wherein said fungal mycelium is free of any stripe, cap or spores;  treating said tissue with an organic solvent solution for a period of time sufficient to permit permeability into the tissue while desiccating the tissue to replace said native moisture with said solvent solution;  removing said tissue from said solution;  pressing said tissue to a minor thickness thereof; and  thereafter drying said tissue, thereby providing a processed mycological biopolymer having a density within a range of 15 pcf to 50 pcf. | Essentially pure mycelium is treated with an organic solvent and then compressed to a target density in the specified range |
| JP7161489B2 | A treated fungal biopolymer material characterized in that it is **composed entirely of hyphae** containing no stalks, caps or spores, and has a **modulus of elasticity of 2-8 psi.** | Pure mycelium with specified elasticity |
| AU2018243372A1 | A processed mycological biopolymer material characterized in being **entirely composed of fungal mycelium** free of any stipe, cap or spores and in having an **elasticity of from 2 to 8 psi**. | Pure mycelium with specified elasticity.  Pending, but granted JP family member has essentially the same features. |
| CA3058212A1 | A processed mycological biopolymer material characterized in being entirely composed of fungal mycelium free of any stipe, cap or spores and in having an **elasticity of from 2 to 8 psi.** | Pure mycelium with specified elasticity.  Pending, but granted JP family member has essentially the same features. |
| EP3599832A4 | see above | see above |
| CN110506104A | see above | see above |
| PCT/US2018/025235 | see above | Pure mycelium with specified elasticity. |
| BR112019020132A8 | Method of Preparation of Mycological Biopolymer Material, characterized in that it comprises the steps of: obtaining a **tissue consisting essentially of fungal mycelium** containing native moisture, in which said fungal mycelium is free of any stipe, covering or spores; treating said fabric with an organic solvent solution for a period of time sufficient to allow permeability in the fabric while desiccating the fabric by **replacing said native moisture with said solvent solution**; removing said tissue from said solution; **pressing** said fabric to a smaller thickness thereof; and then dry said fabric; providing a mycological biopolymer material having a density in a range of 240 kg/m3 to 801 kg/m3. | Pure mycelium with specified density.. |
| US20230013465A1 | A mycological biopolymer material consisting essentially of a fungal mycelium free of any stipe, cap, or spores, wherein the mycological biopolymer material has a **density in a range of 15 pcf to 50 pcf**. | This claim has a different scope from the family members listed above. |
| US11310968B2 | 1. A scaffold structure for growing fungi comprising:  a. a nutrient substrate comprising evenly distributed fungal inoculum;  **b. a porous material positioned away from said nutrient substrate and defining an intermediate layer which does not readily bind with fungal tissue and provides uniform initial conditions of growth, wherein the porous material is microperforated or woven and selected from the group consisting of metal, plastic, and ceramic plate;**  **c. a closed administrable space positioned away from said nutrient substrate and said porous material;**  **d. a first layer of fungal tissue connecting said nutrient substrate to and through said porous material to said administrable space;**  **e. a successive layer of fungal tissue within said administrable space;**  **f. a growth field comprising growth field locations such that growth of said first layer of fungal tissue is directed through said growth field locations so as to create said successive layer of fungal tissue comprising fungal hyphae; and**  **g. a portion of fungal material delaminated from said intermediate layer, the delaminated portion being different from said fungal tissue in that the delaminated portion is chemically or physically altered.** | Method steps b. to g. fairly specific |
| EP3484995A4 | 1. A method of growing fungal material, the method comprising the steps of:  a. providing a nutritive vehicle;  b. growing fungal tissue from said nutritive vehicle, the fungal tissue comprising fungal hyphae;  c. extending fungal tissue growth through a porous material defining an intermediate layer;  d. causing a portion of said fungal tissue extending through the porous material away from said nutritive vehicle to grow into an administrable space, wherein the fungal tissue within said space defines at least one successive fungal material layer;  e. directing a change in the composition or growth pattern of at least one of said fungal hyphae in at least one layer;  f. separating at least a portion of the fungal material from said nutritive vehicle; and  g. altering through physical or chemical means said portion of the fungal material. | see above |
| MX2019386A | Abstract  A method of forming fungal materials and fungal objects from those fungal materials, the method comprising the steps of growing a first fungal tissue in contact with a nutritive vehicle; supplying a porous material in contact with said first fungal tissue; directing growth of said fungal tissue through said porous material such that a portion of said fungal tissue comprises a first fungal material having first fungal hyphae; optionally incorporating composite material; directing a change in the composition or growth pattern of at least some of said first fungal hyphae; separating at least a portion of the first fungal material from said nutritive vehicle; obtaining a second fungal material having second fungal hyphae; and forming a fungal object by encouraging **fused growth between said first fungal material and said second fungal material** and optionally incorporating composite material. | No claims found |
| PCT/US2017/042267 | See EP3484995A4 | See EP3484995A4 |
| US10842089B2 | 1. A fungal growth structure comprising:  a. a nutritive vehicle having a mixture of nutrients and a fungal material distributed throughout;  b. a porous material positioned away from said nutritive vehicle, the porous material being micro-perforated or woven and selected from the group consisting of metal, plastic, and ceramic plate;  c. a closed administrable space positioned away from said nutritive vehicle and said porous material;  d. fungal tissue comprising fungal hyphae having a growth pattern, the growth pattern connecting fungal hyphae from said nutritive vehicle through said porous material to said closed administrable space, wherein the fungal tissue within said closed administrable space defines at least one successive fungal material layer;  e. an intermediate layer defined by said porous material, the intermediate layer which does not readily bind with said fungal tissue, but which provides uniform initial conditions of fungal tissue growth; and  f. a portion of fungal material delaminated from said intermediate layer, the delaminated portion being different from said fungal tissue in that the delaminated portion is chemically or physically altered. | Apart from “scaffold structure“ vs. „fungal growth structure“ a difference to US11310968B2 appear to be variations in the wording. |
| US11013189B2 | 1. A scaffold structure for growing fungi comprising:  a. a substrate having a mixture of nutrients and a fungal material distributed throughout;  b. a **porous material defining an intermediate layer** which does not readily bind with fungal tissue, wherein the porous material is micro-perforated or woven and selected from the group consisting of metal, plastic, and ceramic plate;  c. a closed administrable space positioned away from said substrate and said porous material;  d. a first layer of fungal tissue connecting said substrate to and through said porous material to said administrable space;  e. a successive layer of fungal tissue within said administrable space;  f. a growth field comprising growth field locations such that growth of said first layer of fungal tissue is directed through said growth field locations so as to create said successive layer of fungal tissue, **the successive layer of fungal tissue comprising fungal hyphae having geometries, wherein said fungal hyphae geometries are distorted such that at least one individual hypha extending above the growth field comes** into contact with at least one additional fungal hypha; and  g. a portion of fungal material delaminated from said intermediate layer. | Very specific method including a **porous material defining an intermediate layer** |
| US20220279736A1 | 1. A scaffold structure for growing fungi comprising:  a. a nutrient substrate;  b. a porous material defining an intermediate layer which does not readily bind with fungal tissue, wherein the porous material is selected from the group consisting of metal, plastic, and ceramic plate;  c. a closed administrable space positioned away from said substrate and said porous material;  d. a first layer of fungal tissue connecting said substrate to and through said porous material to said administrable space;  e. a successive layer of fungal tissue within said administrable space;  f. a growth field comprising growth field locations such that growth of said first layer of fungal tissue is directed through said growth field locations so as to create said successive layer of fungal tissue; and  g. a portion of fungal material delaminated from said intermediate layer. | see above |
| US11359174B2 | A method of producing a mycological material comprising the steps of  providing a vessel having a chamber;  loading a substrate of discrete elements inoculated with a filamentous fungus into said chamber;  feeding a **pre-conditioned air stream through said vessel** for diffusion between said discrete elements in said chamber and for a time sufficient for said filamentous fungus to expand a contiguous network of hyphae between and around said discrete elements to form an isotropic inter-particle hyphal matrix; and  **continuing to feed said pre-conditioned air stream through said vessel** for diffusion between said discrete elements and said isotropic inter-particle hyphal matrix for a time sufficient to develop a polarized condition within said vessel wherein air exits said isotropic inter-particle hyphal matrix as a laminar flow into at least one void space within said vessel and to form an extra-particle hyphal matrix extending from said isotropic inter-particle hyphal matrix in the direction of airflow within said at least void space. | Feeding of **air stream between discrete substrate elements** |
| PCT/US2019/047977 | see above | see above |
| US20230056666A1 | A mycological material comprising:  an aerated inter-particle hyphal matrix, the aerated inter-particle hyphal matrix consisting essentially of: a filamentous fungus and a substrate of discrete elements; and  **an aerated extra-particle hyphal matrix growing from the aerated inter-particle hyphal matrix, wherein the aerated extra-particle hyphal matrix has a higher cell volume density than the aerated inter-particle hyphal matrix, a higher anisotropy than the aerated inter-particle hyphal matrix, and a higher hyphal strand thickness than the aerated inter-particle hyphal matrix.** | This device claim is presumably intended to correspond to the method claim above.  However, since the aeration of the process is not a feature of the device, features are used which relate to the particular shape of the hyphae caused by the aeration. |
| AU2019352842A1 | A method of producing a mycological material comprising the steps of providing a vessel having a chamber;  loading a substrate of discrete elements inoculated with a filamentous fungus into said chamber;  **feeding a pre-conditioned air stream** through said vessel for **diffusion between said discrete elements** in said chamber and for a time sufficient for said filamentous fungus to expand a contiguous network of hyphae between and around said discrete elements to form an isotropic inter-particle hyphal matrix;  and continuing to feed said pre-conditioned air stream through said vessel for diffusion between said discrete elements and said isotropic inter-particle hyphal matrix for a time sufficient to develop a polarized condition within said vessel wherein air exits said isotropic inter-particle hyphal matrix as a laminar flow into at least one void space within said vessel and **to form an extra-particle hyphal matrix extending from said isotropic inter-particle hyphal matrix in the direction of airflow within said at least void space**. | Feeding of **air stream between discrete substrate elements** |
| CA3113935A1 | See AU2019352842A1 above. | See AU2019352842A1 above. |
| EP3860370A4 | See AU2019352842A1 above. | See AU2019352842A1 above. |
| **!!!**  US11277979B2 | A process of growing a mycological biopolymer material, comprising the steps of  providing a tool defining a cavity therein with an opening into said cavity;  packing said cavity of the tool with nutritive substrate and a fungus;  placing **a lid on said tool to cover said cavity, said lid having only one outlet therein** defining a void space open to fresh air;  allowing said fungus to grow mycelium within said cavity and to allow the mycelium to respirate within said cavity thereby producing carbon dioxide while colonizing the nutritive substrate;  **allowing the produced carbon dioxide to diffuse out of said outlet in said lid to create a gradient of carbon dioxide; and**  **allowing the mycelium to grow along said gradient to fill said void space without producing a stipe, cap or spore therein and to produce a mycelium biopolymer in said void space.** | Although the characteristic features sound carefully phrased and the description suggests that a mycelium material is to be produced, the combination of features in claim 1 reads as if it were realised by a bucket with a broken or loosely placed lid with substrate and mycelium for growing edible mushrooms (and therefore possibly not new).  But as long as the patent is not challenged, it remains in force. |
| **!!!**  US20220290199A1 | An apparatus for growing a mycological biopolymer, comprising:  a tool defining a cavity and an opening into the cavity;  a nutritive substrate and a fungus, the nutritive substrate inoculated with fungal mycelia and positioned within the cavity, wherein growth of the mycelia within the cavity produces carbon dioxide;  a lid configured to fit on the tool to seal the cavity, the lid having only one lid outlet therein defining a void space open to fresh air,  wherein the lid outlet is configured to allow the carbon dioxide to diffuse out of the tool to create a gradient of carbon dioxide, and  wherein the void space is configured to provide the mycelia with space to grow along the gradient without producing a stipe, cap or spore therein | Apparatus claim corresponding to the method claim of US11277979B2 |
| US10945382B2 | 1. A **self-contained soil module**, the self-contained soil module comprising:  a biodegradable outer frame forming an outer surface of the self-contained soil module comprising a top side, a bottom side, and one or more side walls, wherein the biodegradable outer frame is constructed from at least a mycelium sheet, the mycelium sheet grown from a mycelium substrate mixture, the mycelium substrate mixture configured to grow such that the mycelium substrate mixture fully forms at least one of the top side, the bottom side, and the one or more side walls;  **a soil composition disposed within the biodegradable outer frame; and**  **an inner layer comprising a biodegradable wrapping disposed within the biodegradable outer frame, the biodegradable wrapping comprising an upper sheet and a lower sheet, wherein the upper sheet and the lower sheet are situated in parallel planes;**  **at least one plant seed of at least one type of plant disposed between the upper sheet and the lower sheet; and**  **a grid structure disposed on the bottom side, the grid structure in contact with the one or more side walls.** | The mycelial composite is supplemented by several elements that are not directly related to the mycelial composite (**soil, layers with seeds, lattice**). |
| US11102938B2 | A **method of manufacturing a self-contained soil module**, the method comprising:  growing at least one mycelium sheet;  cutting the at least one mycelium sheet into a bottom layer, a top layer, and one or more side walls;  forming the bottom layer and the one or more side walls into a shape of a hollow container;  binding the bottom layer and the one or more side walls using a biobased mastic;  forming a soil composition; forming a soil packet comprising the steps of:  placing the soil composition on top of a biodegradable wrapping;  placing the biobased mastic along at least one edge of the biodegradable wrapping;  sealing the soil packet by adhering the at least one edge of the biodegradable wrapping to a second edge of the biodegradable wrapping, such that the soil composition is disposed within the biodegradable wrapping;  compressing the soil packet into the shape of the hollow container;  placing the soil packet contained within the sealed biodegradable wrapping into the hollow container;  placing and sealing the mycelium sheet top layer onto the one or more side walls with the biobased mastic; and  injecting, at any of the preceding steps, at least one seed of at least one type of plant into at least one of the bottom layer, the top layer, the one or more side walls, or the soil packet. | Apparatus claim corresponding to the method claim of US10945382B2 |
| US11015059B2 | A composite mycelium material, comprising:  a. a cultivated mycelium material comprising one or more masses of branching hyphae, wherein the **one or more masses of branching hyphae is disrupted;** and  b. a **bonding agent** selected from the group consisting of a vinyl acetate-ethylene (VAE) copolymer, a vinyl acetate-acrylic copolymer, a polyamide-epichlorohydrin resin (PAE), a copolymer, transglutaminase, citric acid, genipin, alginate, gum arabic, latex, a natural adhesive, and a synthetic adhesive. | Combination of mycelium material and **bond material** |
| US11891514B2 | A composite mycelium material, comprising:  a. a cultivated mycelium material comprising one or more masses of branching hyphae, wherein the one or more masses of branching hyphae is disrupted; and  b. a **bonding agent**; and  c. a **dye**. | Different scope of protection than US11015059B2: Bonding agent not specified, but additional **die**. |
| SG11202112275VA | Claims not found | Claims may possibly be similar to CA3137693A1 and the others. |
| CA3137693A1 | A composite mycelium material, comprising:  a. a cultivated mycelium material comprising one or more masses of branching hyphae, wherein the one or more masses of branching hyphae is **disrupted**; and  b. a **bonding agent**. | Broader than US11015059B2, but still pending. |
| MX2021014233A | Claims not found | Claims may possibly be similar to CA3137693A1 and the others. |
| TW202112943A | A composite mycelium material, which comprises: a. Cultivated mycelium material containing one or more **clumps** of branched hyphae, where the one or more clumps of branched hyphae are disrupted; and b. **Adhesives**. | Broader than US11015059B2, but still pending. |
| CN114127278A | 1.A composite mycelium material comprising:  a. Cultivated mycelial material comprising one or more **clumps** of branched hyphae, wherein the one or more clumps of branched hyphae are disrupted; and  b. **Adhesive**. | Differences to CA3137693A1 and the other pending applications appear to be due to translation. |
| JP2022534025A | a. a cultured mycelial material comprising one or more **clumps** of branched mycelia, wherein said one or more clumps of branched mycelia are disrupted; b. A composite mycelial material comprising a **binding agent**. | It is not quite clear whether this machine translation of the Japanese claim 1 is complete. |
| AU2020279832A1 | A composite mycelium material, comprising:  a. a cultivated mycelium material comprising one or more masses of branching hyphae, wherein the one or more masses of branching hyphae is **disrupted**; and  b. a **bonding agent**. | Broader than US11015059B2, but still pending. |
| EP3973055A4 | A composite mycelium material, comprising:  a. a cultivated mycelium material comprising one or more masses of branching hyphae, wherein the one or more masses of branching hyphae is **disrupted**; and  b. a **bonding agent**. | Broader than US11015059B2, but still pending. |
| KR20220027075A | A composite mycelium material comprising:  a. a cultured mycelium material comprising at least one **mass of branching hyphae**, wherein the at least one mass of branching hyphae is disturbed; and  b. A composite mycelium material comprising a **binder**. | Differences to CA3137693A1 and the other pending applications appear to be due to translation |
| **!!!**  EP2094856B1 | A method of making a composite material characterized in the steps of  forming an inoculum including a preselected fungus;  forming a mixture of a substrate of discrete particles and a nutrient material, said nutrient material being capable of being digested by said fungi;  adding said inoculum to said mixture; and  allowing said fungus to digest said nutrient material in said mixture over a period sufficient to grow hyphae and to allow said hyphae to form a network of interconnected mycelia cells through and around said discrete particles thereby bonding said discrete particles together to form a **self-supporting composite material.** | Even the heating is missing here, and it was nevertheless granted.  An opposition was filed against the patent on 29 December 2016, and the preliminary opinion of the board was that the subject matter of claim 1 (and others) **lacks novelty** over D1, D7, and D9 (see below), but the patent was upheld unamended in a decision dated 10 May 2018 due to a withdrawal of opposition.  Cited prior art included: US5074959A (D1);  R Rush Wayne: Growing Mushrooms the Easy Way – Home Mushroom Cultivation with Hydrogen Peroxide – Volume I, 1September 2001 (2001-09-01), XP055337498, retrieved from the Internet: <URL:http:/jontrot.free.fr/champignons/culture-eau-oxygenee-Vols1-2new.pdf> (D7),  Philip Ross: “PURE CULTURE 1997-present”, 1 January 2016 (2016-01-01), XP055337515 (D9),  and others |
| US9485917B2 | A method of making a composite material comprising the steps of  forming an inoculum including a preselected fungus;  forming a mixture of a **substrate of discrete particles** and a nutrient material, said nutrient material being capable of being digested by said fungi;  adding said inoculum to said mixture,  allowing said fungus to digest said nutrient material in said mixture over period sufficient to grow hyphae and to allow said hyphae to form a network of interconnected mycelia cells through and around said discrete particles thereby bonding said discrete particles together to form a **self-supporting composite material**; and  wherein at least one of said inoculum and said mixture includes water and which further comprises the step of **heating the formed self-supporting composite material to a temperature sufficient to kill said fungus**. | Even if it may seem that heating is relevant to distinguish the composite material from a profane inoculated substrate for **growing edible mushrooms**: This does not appear to be the case for some patent offices, e. g., EP (see comment on EP209485 below) |
| IL199315A | No claims found | See general comments on family members |
| US8999687B2 | A panel comprising  a self-supporting composite material formed of a substrate of discrete particles selected from the group consisting of straw, hemp, wool, recycled sawdust and cotton, and a network of interconnected mycelia cells produced from the group consisting of at least one of Agrocybe brasiliensi, Flammulina velutipes, Hypholoma capnoides, Hypholoma sublaterium, Morchella angusticeps, Macrolepiota procera and Coprinus comatus and extending through and around said discrete particles and bonding said discrete particles together,  said composite material having at least one exterior surface of a predetermined length and a thickness less than said length; and  a veneer material bonded to said exterior surface and wherein said panel is a structural insulating panel. | Specific **materials** for particles and mycelium, and product specified to be a **panel** |
| US10525662B2 | A product comprising  a self-supporting composite material including a substrate of discrete particles and a network of interconnected mycelia cells extending through and around said discrete particles and bonding said discrete particles together; and  **at least one element embedded in said self-supporting composite material** | Object embedded in mycelium |
| US9801345B2 | A self-supporting composite material comprising a plurality of layers of engineered substrate wherein each said layer including a plurality of discrete particles and said mycelia cells extend through and around said plurality of discrete particles to bond said particles together into a coherent whole, and a **network of interconnected mycelia cells extending through and bonding said layers together.** | Connected layers |
| US9795088B2 | A method of making a **self-supporting** composite material comprising the steps of  creating an engineered substrate comprised of a nutrient source and at least one of discrete particles and fibers;  disposing the substrate within an enclosure in an amount to fill said enclosure;  inoculating the substrate within the enclosure with an inoculum containing a desired fungi strain;  growing the desired fungi strain through the engineered substrate within the enclosure for a time sufficient for said fungal strain to digest said nutrient source, to grow hyphae and to allow said hyphae to form a network of interconnected mycelia cells through and around said at least one of discrete particles and fibers thereby bonding said at least one of discrete particles and fibers together to form a cohesive whole with a shape matching the internal shape of said enclosure;  **compressing a tooling piece with at least one protrusion into at least one face of the engineered substrate during growth of said fungi strain to forcefully mold a corresponding feature to said protrusion into the engineered substrate and resultant cohesive whole**; and  thereafter removing the cohesive whole from said enclosure and drying the cohesive whole. | Forming of a **depression** in substrate during mycelium growth |
| JP5740492B2 | Forming an inoculum comprising a preselected fungus,  Forming a mixture of individual particle culture medium and the nutrient material digestible by the fungus;  Placing the mixture in a housing;  Adding the inoculum to the mixture in the housing;  Allowing the fungus to digest the nutrient material in the mixture for a period of time sufficient to grow to a fruiting body **that fills the housing and extends outside the culture medium**; and removing the fruiting body from the housing | Shaping of composite by housing |
| **!!!**  JP5457194B2 | Forming an inoculum comprising a preselected fungus,  Forming a mixture of the individual particle culture medium and the nutrient material digestible by the fungus,  Adding said inoculum to said mixture; and said fungus for a period of time sufficient to grow mycelium and to form a network of interconnected mycelium cells between and around said individual particles. Digesting the nutrient material in the mixture to bind the individual particles together to form a **self-supporting composite material**. | Minimum set of features, see comments on EP2094856B1 |
| IL234585A | Title:  Panels comprising a self-supporting composite material and a veneer material | No claims found, but since the title has been adjusted, the claims could be aimed at such an object. |
| IL234584B | Title:  Self-supporting composite material comprising a substrate of discrete particles and a network of interconnected mycelia cells | No claims found, but since the title has been adjusted, the claims could be aimed at such an object. |
| **!!!**  US10589489B2 | A **self-supporting** composite material comprising  a substrate of discrete particles; and  a network of interconnected mycelia cells extending through and around all of said discrete particles to fully colonize said substrate and bond said discrete particles together. | Minimum set of features, see comments on EP2094856B1 |
| **!!!**  US10583626B2 | A method of making a composite material comprising the steps  of  forming an inoculum including a preselected fungus;  forming a mixture of a substrate of discrete particles and a nutrient material, said nutrient material being capable of being digested by said fungi;  adding said inoculum to said mixture; and  allowing said fungus to digest said nutrient material in said mixture over a period sufficient to grow hyphae and to allow said hyphae to form a network of interconnected mycelia cells through and around all of said discrete particles thereby bonding all of said discrete particles together with said network to form a **self-supporting composite material**. | Same features as EP2094856B1 |
| US11932584B2 | A method of forming a product comprising  providing a **three-dimensional lattice** having at least two grids oriented orthogonally to each other;  coating the lattice with a **mixture of starch and water**;  thereafter placing the lattice in a bed of inoculum containing **Pleurotus ostreatus** in a nutrient carrier;  thereafter stimulating mycelium growth over and through the grids of the lattice to produce a dense network of hyphae; and  allowing the hyphae to interweave over time to produce a mat of thickly formed mycelia on the lattice. | Three-dimensional lattice overgrown by Pleurotus ostreatus |
| AU2007333545B2 | A method of making a composite material characterized in the steps of  forming an inoculum including a preselected fungus;  forming a mixture of a substrate of discrete particles and a nutrient material, said nutrient material being capable of being digested by said fungi;  adding said inoculum to said mixture;  growing the inoculated mixture in an enclosure having a volume denoting the final form of the composite material to be made;  allowing said fungus to digest said nutrient material in said mixture over a period sufficient to grow hyphae and to allow said hyphae to form a network of interconnected mycelia cells through and around said discrete particles within the enclosure thereby bonding said discrete particles together to form a **self-supporting composite material conforming to the volume of the enclosure.** | Enlosure is additionally specified.  Not very limiting, though, since also in the prior art (e. g., for forming edible mushrooms) the inoculated substrate will typically be arranged in some kind of enclosure. |
| CA2672312C | see EP856 | see EP856 |
| CN101627127B | see EP856 | see EP856 |
| KR102001771B1 | (a) combining coffee grounds and sawdust in a weight ratio of 4: 6 to 6: 4, supplying water to adjust the water content of the mixture to 65% to 70%, and then forming a solid medium, and sterilizing for 1 hour in a high pressure reactor. (B) taking out the solid medium sterilized in step (a) and cooling it; (c) **introducing mushroom seedlings into a liquid potato extraction medium (PDB, Potato Dextrose Broth) to produce mushroom liquid seedlings; step**; (d) inoculating the mushroom liquid spawn prepared in step (c) into a solid medium using a spawn inoculator; (e) the resultant of step (d) at a temperature of 20 ° C to 28 ° C and a humidity of 65% (F) after step (e), after drying for 3 hours at 130 ° C. using a vacuum dryer to produce the final product, and cooling the dried medium using a compressor, Before the step (d), the seed inoculator and the inoculation place before the seed inoculation are further sterilized with a disinfectant containing ethanol, and after the seed inoculation further includes a sterilization step of removing internal bacteria using a UV irradiator installed inside the inoculation chamber. In step (a), when the **coffee grounds and sawdust are mixed, more flour is added**. At this time, the amount of flour is added in 10 to 20% by weight based on the total weight of the solid medium. Just before the fruiting body After full growth, further culture step is further cultured for about 5 to 7 days, step (e) is to cover the solid medium and incubated at a temperature of 20 ℃ to 28 ℃ to avoid invasion of bacteria from the outside, After a certain period of time after the cultivation of the mycelia, the inoculated Pleurotus eryngii comprising the step of inverting the aeration so that the culture can be uniformly cultured in a solid medium, the operation according to the manufacturing process of steps (a) to (f) Each cultivation room, which is a space made up, is selectively installed with a plurality of sensors including a temperature sensor, a humidity sensor, and an illuminance sensor, and in each cultivation room, when an inappropriate sensing value is detected, which is out of a predetermined error range from a preprogrammed standard value. There is to turn on the lamp located on one side of each cultivation room, or to send an alarm sound to the manager's smart device so that the operator can recognize. The method of eco-functional materials using the coffee grounds are included. | Specific information for the process:  Materials, temperatures, humidity, etc. |
| US8313939B2 | A method of making a molded part, comprising:  forming a mixture of a fungal inoculum, a nutrient source for the fungal inoculum, and a liquid;  injecting the mixture into a first mold cavity;  sealing the first mold cavity against a second mold cavity;  **growing live mycelium from the fungal inoculum to fill the first and second mold cavities to form a first molded part;**  curing the live mycelium to terminate further growth;  separating the first mold cavity and the second mold cavity;  injecting a mycelium slurry over the first molded part in the first mold cavity;  sealing the first mold cavity against a third mold cavity;  **growing live mycelium from the mycelium slurry to form a second molded part over the first molded part; and**  curing the live mycelium of the second molded part to terminate further growth and **develop a dual mycelium molded part made up of the first molded part and the second molded part.** | Two separate growth and therefore moulding processes |
| US8298809B2 | A method of making a **hardened elongate structure**, comprising:  growing mycelium for a period between 1 and 15 days to produce a live mycelium mat having a thickness between approximately 0.125 inches (0.3175 cm) and 2.0 inches (5.08 cm) and having branching hyphae;  **layering the live mycelium mat to form an elongate multi-layered structure**;  **allowing the hyphae to grow inward into the multi-layered structure such that the hyphae are interwoven throughout the multi-layered structure**; and  curing the multi-layered structure by heating the structure to a temperature of at least 150 degrees Fahrenheit for a period of at least one day to terminate mycelium growth and form a hardened elongate structure. | **Reshaping** of the mycelial mat during a growth process to form an **elongated structure**. |
| US8298810B2 | A method of making an injection molded part, comprising:  combining a fungal inoculum with a liquid and a nutrient source to form a mixture;  inserting a coverstock into a hydraulic press injection mold having a closed mold cavity;  injecting the mixture into the closed mold cavity of the hydraulic press injection mold through an injection port;  **growing live mycelium from the mixture that fills the closed mold cavity and physically couples with the coverstock; and**  **heating the live mycelium to terminate further growth and develop an injection molded part made of mycelium and the coverstock for use in a vehicle interior**. | Composite material with integrated injection mould |
| US8283153B2 | A method of making a molded part, comprising:  mixing an aggregate with a fungal inoculums to form a mixture;  evenly distributing **nanoparticles** throughout the mixture;  inserting the mixture into a mold cavity;  growing live mycelium to fill the mold cavity; and  curing the live mycelium to terminate further growth of the molded part. | Nanoparticles during the moulding process |
| US8227224B2 | A method of making a molded part, comprising:  inserting a fungal inoculum and a mixture comprising a liquid and a nutrient for the fungal inoculum into a mold cavity;  **inserting a portion of a mechanical device into the mold cavity such that a portion of the mechanical device is exposed by not being inserted in the mold cavity**;  growing the fungal inoculum into **live mycelium that operably couples with the portion of the mechanical device inserted in the mold cavity**, and such that the mycelium does not couple to exposed portion of the mechanical device; and  heating the mycelium to terminate further growth and develop a molded part made of mycelium and the mechanical device. | Partial embedding of a mechanical device for operative coupling |
| US8227225B2 | A method of making a mycelium structure, comprising:  dissolving a **soluble plastic film having insoluble polymer particles in a liquid to form a solution of polymer particles**;  combining the solution of polymer particles with a fungal inoculum and a nutrient source for the inoculum to form a mixture;  growing a live **mycelium network that bonds with the polymer particles to form a plasticized structure**; and  terminating growth. | Combination of the mycelium nutrient growth solution with a polymer particle solution, wherein the mycelium is adapted to bond with the polymer |
| US8227233B2 | A method of making a **foamed mycelium structure,** comprising:  providing a fungal inoculum having a fungus capable of growing hyphae;  adding the fungal inoculum to a liquid and a nutrient source for the inoculum to form a **slurry**;  placing the slurry in a reaction vessel having an agitation device;  agitating the slurry in the presence of at least one select **gas to create gas bubbles in the slurry;**  allowing the fungus to grow a live mycelium network **through and around the gas bubbles**; and  terminating growth. | Foamed mycelium structure |
