# Supplementary material for: Patent landscape analysis for materials based on fungal mycelium: a guidance report on how to interpret the current patent situation
Source: Fungal Biol Biotechnol. 2024 Aug 10;11:11. doi: 10.1186/s40694-024-00177-2 (PMC11316976; doi:10.1186/s40694-024-00177-2)
Supplement: Supplementary file 2 — Additional file 2. Features of the family member patents of EP2094856B1 (EP856). [file 40694_2024_177_MOESM2_ESM.docx]

Additional File 2. Features of the family member patents of EP2094856B1 (EP856)

| Patent Number | Features of claim 1 | Comment |
| --- | --- | --- |
| EP2094856B1 | A - A method of making a composite material characterized in the steps of  B - forming an inoculum including a preselected fungus;  C - forming a mixture of a substrate of discrete particles and a nutrient material, said nutrient material being capable of being digested by said fungi;  D - adding said inoculum to said mixture; and  E - allowing said fungus to digest said nutrient material in said mixture over a period sufficient to grow hyphae and  F - to allow said hyphae to form a network of interconnected mycelia cells through and around said discrete particles  G - thereby bonding said discrete particles together to form a self-supporting composite material. | Minimal set of features |
| US10583626B2 | A to G | Same as EP856 |
| US9485917B2 | H - wherein at least one of said inoculum and said mixture includes water and  I - which further comprises the step of **heating** the formed self-supporting composite material to a temperature sufficient to kill said fungus. | Additional features H and I |
| US10589489B2 | J - A self-supporting composite material comprising  K - a substrate of discrete particles; and  L - a network of interconnected mycelia cells extending through and around all of said discrete particles to fully colonize said substrate and bond said discrete particles together. | Device claim with minimal set of features.  Correspondences:  G ≈ J  K ≈ part of C  L ≈ F |
| US8999687B2 | M - A panel comprising  N - a self-supporting composite material formed of a substrate of discrete particles selected from the group consisting of straw, hemp, wool, recycled sawdust and cotton, and a network of interconnected mycelia cells produced from the group consisting of at least one of Agrocybe brasiliensi, Flammulina velutipes, Hypholoma capnoides, Hypholoma sublaterium, Morchella angusticeps, Macrolepiota procera and Coprinus comatus and extending through and around said discrete particles and bonding said discrete particles together,  said composite material having at least one exterior surface of a predetermined length and a thickness less than said length; and  O - a veneer material bonded to said exterior surface and  P - wherein said panel is a structural insulating panel. | Substantially different from EP856 and with fairly specific set of features |
| US10525662B2 | Q - A product comprising  J, K, L; and  R - at least one element embedded in said self-supporting composite material | Additional features Q and R |
| US9801345B2 | J  S - a plurality of layers of engineered substrate  T - wherein each said layer including a plurality of discrete particles and  L’ - said mycelia cells extend through and around said plurality of discrete particles to bond said particles together into a coherent whole, and  U - a network of interconnected mycelia cells extending through and bonding said layers together. | Feature L’ differs minimally from feature L; additional features S, T, and U. |
| US9795088B | A’ - A method of making a self-supporting composite material comprising the steps of  V - creating an engineered substrate comprised of a nutrient source and at least one of discrete particles and fibers;  W - disposing the substrate within an enclosure in an amount to fill said enclosure;  D’ - inoculating the substrate within the enclosure with an inoculum containing a desired fungi strain;  E’ - growing the desired fungi strain through the engineered substrate within the enclosure for a time sufficient for said fungal strain to digest said nutrient source,  F’ - to grow hyphae and to allow said hyphae  G’ - to form a network of interconnected mycelia cells through and around said at least one of discrete particles and fibers thereby bonding said at least one of discrete particles and fibers together  X - to form a cohesive whole with a shape matching the internal shape of said enclosure;  Y - compressing a tooling piece with at least one protrusion into at least one face of the engineered substrate during growth of said fungi strain to forcefully mold a corresponding feature to said protrusion into the engineered substrate and resultant cohesive whole; and  Za - thereafter removing the cohesive whole from said enclosure and  Zb - drying the cohesive whole. | Same basic method as EP856, but with additional features V, W, X, Y |
| JP5740492B2 | B  C’ - Forming a mixture of individual particle culture medium and the nutrient material digestible by the fungus;  W’’ - Placing the mixture in a housing;  D’’ - Adding the inoculum to the mixture in the housing;  E’’ - Allowing the fungus to digest the nutrient material in the mixture for a period of time sufficient to grow to a fruiting body  Zc - that fills the housing and extends outside the culture medium; and  Za - removing the fruiting body from the housing. | Correspondences:  Zb ≈ E  Zc ≈ X  It is assumed that the term “fruiting body” in the translation refers to the network of hyphae, not to what is commonly understood as “fruiting body” in the context of fungi |
| JP5457194B2 | B to G | Slight differences in the wording may be due to translation |
| US11932584B2 | Zd - A method of forming a product comprising  Ze - providing a three-dimensional lattice having at least two grids oriented orthogonally to each other;  Zf - coating the lattice with a mixture of starch and water;  Zg - thereafter placing the lattice in a bed of inoculum containing Pleurotus ostreatus in a nutrient carrier;  Zh - thereafter stimulating mycelium growth over and through the grids of the lattice to produce a dense network of hyphae; and  Zj - allowing the hyphae to interweave over time to produce a mat of thickly formed mycelia on the lattice. | Substantially different from EP856 and with fairly specific set of features |
| AU2007333545B2 | A to D  Zj - growing the inoculated mixture in an enclosure having a volume denoting the final form of the composite material to be made;  E - allowing said fungus to digest said nutrient material in said mixture over a period sufficient to grow hyphae and to allow said hyphae to form a network of interconnected mycelia cells through and around said discrete particles  Zk - within the enclosure thereby bonding said discrete particles together to form a self-supporting composite material conforming to the volume of the enclosure. | Additional features Zj and Zk specifying an enclosure |
| CA2672312C | A to G | Minimal set of features |
| CN101627127B | A to G | Minimal set of features |
